# Generation, validation, and benchmarking of a commercial independent Monte Carlo calculation beam model for multi-target SRS

**DOI:** 10.1016/j.zemedi.2023.08.004

**Published:** 2023-09-14

**Authors:** Justus Adamson, Brett G. Erickson, Chunhao Wang, Yunfeng Cui, Markus Alber, John Kirkpatrick, Fang-Fang Yin

**Affiliations:** a Duke University Medical Center, Department of Radiation Oncology, Durham, NC, USA; b Scientific RT GmbH, Munich, Germany

**Keywords:** Monte Carlo, Radiosurgery, TG-219

## Abstract

**Background:**

Dosimetric validation of single isocenter multi-target radiosurgery plans is difficult due to conditions of electronic disequilibrium and the simultaneous irradiation of multiple off-axis lesions dispersed throughout the volume. Here we report the benchmarking of a customizable Monte Carlo secondary dose calculation algorithm specific for multi-target radiosurgery which future users may use to guide their commissioning and clinical implementation.

**Purpose:**

To report the generation, validation, and clinical benchmarking of a volumetric Monte Carlo (MC) dose calculation beam model for single isocenter radiosurgery of intracranial multi-focal disease.

**Methods:**

The beam model was prepared within SciMoCa (ScientificRT, Munich Germany), a commercial independent dose calculation software, with the aim of broad availability via the commercial software for use with single isocenter radiosurgery. The process included (1) definition & acquisition of measurement data required for beam modeling, (2) tuning model parameters to match measurements, (3) validation of the beam model via independent measurements and end-to-end testing, and finally, (4) clinical benchmarking and validation of beam model utility in a patient specific QA setting. We utilized a 6X Flattening-Filter-Free photon beam from a TrueBeam STX linear accelerator (Siemens Healthineers, Munich Germany).

**Results:**

In addition to the measured data required for standard IMRT/VMAT (depth dose, central axis profiles & output factors, leaf gap), beam modeling and validation for single-isocenter SRS required central axis and off axis (5 cm & 9 cm) small field output factors and comparison between measurement and simulation of backscatter with aperture for jaw much greater than MLCs. Validation end-to-end measurements included SRS MapCHECK in StereoPHAN geometry (2%/1 mm Gamma = 99.2% ± 2.2%), and OSL & scintillator measurements in anthropomorphic STEEV phantom (6 targets, volume = 0.1–4.1cc, distance from isocenter = 1.2–7.9 cm) for which mean difference was −1.9% ± 2.2%. For 10 patient cases, MC for individual PTVs was −0.8% ± 1.5%, −1.3% ± 1.7%, and −0.5% ± 1.8% for mean dose, D_95%_, and D_1%_, respectively. This corresponded to custom passing rates action limits per AAPM TG-218 guidelines of ±5.2%, ±6.4%, and ±6.3%, respectively.

**Conclusions:**

The beam modeling, validation, and clinical action criteria outlined here serves as a benchmark for future users of the customized beam model within SciMoCa for single isocenter radiosurgery of multi-focal disease.

## 1 Introduction

Patients with brain metastases often undergo treatment using stereotactic radiosurgery (SRS) [1]. Simultaneous treatment to multiple metastases using a single isocenter is a treatment technique that has recently grown in prevalence with various reported treatment planning strategies [2], [3], immobilization and quality assurance procedures [4], [5], as well as clinical outcomes [6], [7], [8]. Single isocenter techniques offer the advantage of improved treatment efficiency for multiple metastases but with ongoing challenges specifically for dosimetry and plan verification.

Regarding challenges of accurate dosimetry for single isocenter SRS, because targets are not necessarily located at isocenter, the accuracy of beam data (small field output factors, depth dose curves, profiles, leaf modeling) is essential not only at the central axis, but also at off-axis locations. In some instances this has led to institutions having difficulty commissioning a multi-target technique that is robust for all geometries and off axis distances [9]. In addition, the robustness of single isocenter Volumetric Modulated Arc Therapy (VMAT) to beam modeling errors is dependent on plan complexity and modulation; thus the patient specific QA procedure must assure that each plan does not fall outside the capabilities of the beam model [10].

Regarding challenges with plan verification, the isolated small high dose volumes located throughout the anatomy results in a uniquely complicated measurement for patient specific pre-treatment QA. Pretreatment validation measurements should ideally validate the dosimetry for each individual target with sufficient spatial resolution; provided the small target volume of many SRS targets, this requires a finer spatial resolution than would be sufficient for standard Intensity Modulated Radiotherapy (IMRT) and VMAT. Solutions that are currently available include detectors that have sufficiently high resolution but are not comprehensive with a single measurement such as film [11], [12], and SRS specific diode arrays such as the SRS MapCHECK device [13] (Sun Nuclear Corporation, Melbourne FL) and the myQA SRS device [14] (IBA, Louvain-La-Neuve, Belgium); as well as detectors that provide a more comprehensive volumetric measurement but with less than ideal spatial resolution for SRS [15], [16]. Portal dosimetry based verification techniques are capable of comprehensive and efficient verification of the delivered fluence at high spatial resolution [17], [18], [19], [20]. However, these devices do not verify the performance of the beam modeling & dose calculation with respect to the plan of interest, and in some cases integrate across all gantry angles for modulated arcs, thus lacking the angular resolution necessary for dosimetric backprojection. 3D dosimetry solutions offer both high resolution and comprehensive measurement [21], [22], [23], but are typically reserved for commissioning and end-to-end testing rather than patient specific pre-treatment QA due to cost, inefficiency of measurement, and need for specialized expertise, equipment, and analysis software. Thus, there remains a need for a patient specific quality assurance verification in this context that is sufficiently sensitive, comprehensive, and efficient.

One quality assurance check that nicely complements the limitations of the pre-treatment QA measurement is a rigorous and volumetric independent dose calculation. Patient specific pre-treatment QA traditionally consists of both an independent dose calculation [24] and a pre-treatment measurement [25]. While the independent dose calculation has traditionally consisted of a simple (hand) calculation to a single point, more sophisticated calculation algorithms with volumetric dose calculation have recently been implemented into independent calculation software from numerous vendors. Recommendations regarding use of volumetric independent calculation software in conjunction with intensity modulated plans were recently published [24], but lack reported experience with clinical workflows and DVH based action / tolerance criteria. For SRS specifically, Calvo-Ortega et al. recently applied a Monte Carlo independent dose calculation for HyperArc plans and provided Gamma Index passing rates for 35 plans (60 targets) [26]. Hillman et al. revised the commercial Mobius 3D convolution superposition algorithm for use with radiosurgery planning, achieving agreement within 3% for field sizes of ≥2 × 2 cm^2^ and noting the need for further improvements for smaller field sizes [27]. In this study, we apply a previously existing commercial independent dose calculation software, SciMoCa (ScientificRT, Munich Germany) [28], for use with single isocenter treatment planning for multi-focal SRS. Specifically, in collaboration with the vendor we customized the Monte Carlo dose calculation algorithm beam model and verify its accuracy for validation of SIMT SRS plans. The aim for this beam model is widespread availability for potential future users via deployment as a customizable option within the software for use with single isocenter radiosurgery.

## 2 Methods

### 2.1 Overview

We recently commissioned a Monte Carlo dose calculation beam model customized for use with single isocenter multitarget SRS within a commercially available independent dose calculation software, SciMoCa (ScientificRT, Munich Germany) [28]. This process included (1) definition & acquisition of measurement data required for beam modeling, (2) tuning model parameters to match measurements, (3) validation of the beam model via independent measurements and end-to-end testing, and finally, (4) clinical benchmarking and validation of beam model utility in a patient specific QA setting. These steps are elaborated in subsequent sections.

### 2.2 Commissioning

#### 2.2.1 Treatment planning system

The Treatment Planning System (TPS) utilized for this study was ARIA External Beam Planning version 15.6 (Siemens Healthineers, Munich Germany). We used a 6X Flattening Filter Free (FFF) beam from a TrueBeam STX linear accelerator (Siemens Healthineers, Munich Germany) modeled using the Anisotropic Analytical Algorithm (AAA) version 13.6.23. The AAA beam model was previously commissioned for radiosurgery treatment of multiple metastases, with accuracy of the beam model validated and reported previously [21]. All calculations were carried out with a 1 mm isotropic dose grid.

The Monte Carlo algorithm within the SciMoCa independent dose calculation software has been described previously [28], [29], [30], and utilizes a source model composed of various virtual sources (primary, primary collimator scatter, head & flattening filter scatter, backscatter, electron contamination). Source properties are modified [31] from a generic linear accelerator model using feedback from BEAMnrc simulations to achieve a specific treatment machine model. At the dose calculation stage, material property is derived from CT images and grouped into material categories (such as “lung-like,” “soft-tissue-like,” and “bone-like,”) with variable density. All calculations were performed using a 1 mm isotropic voxel size in order to correspond with the TPS dose grid.

#### 2.2.2 Beam data acquisition, modeling, & validation

The beam modeling process of the SciMoCa algorithm was carried out with the aim of matching the beam model to measured data, rather than matching the beam data from the primary treatment planning system. During the beam modeling, the following model traits were tuned to specific measurements in the following order:

1. The primary photon spectrum derived from depth dose curves, predominantly of fields between 3 × 3 cm^2^ to 10 × 10 cm^2^.
2. The calibration of collimator jaw positions derived from in-plane and cross-plane profiles.
3. The calibration of the primary focal spot derived from jaw-defined small fields of about 1 × 1–2 × 2 cm^2^, combined with cross-profiles obtained with a small sensitive volume detector.
4. The calibration of the MLC leaf positions and inter-leaf-leakage derived from dosimetric leaf gap measurements (BrainLab, Munich Germany), where the transmission value translates into an MLC-specific average inter-leaf-gap.
5. The off-axis behavior of the energy fluence derived from cross-profiles of large fields at depth of 5 cm.

Further invariable model traits that are relevant for single isocenter SRS are MLC leaf geometries and backscatter properties of collimator jaws and leaves. The leaves are simulated as 3D objects with curved tips, stepped sides and adjustable inter-leaf-gap to account for manufacturing tolerances. The backscatter properties are derived from detailed accelerator head simulations and specific measurements on a variety of systems.

A set of measured beam data was used for preparing the Monte Carlo beam model, after which an independent set of measured data was used as validation of the beam model. This process included validating increasingly challenging scenarios, culminating in end-to-end testing. The data for this process is summarized in Table 1, and included general inline and crossline profiles, depth dose curves, output factors, and dosimetric leaf gap measurements. Input data that was specific to a single isocenter VMAT SRS approach included small field profiles and MLC field output factors at central axis, and small field profiles and MLC output factors at off axis locations. As outlined in Table 1, central axis and off axis output factors were measured using a number of detectors, with correction factors applied based on the guidelines on small field dosimetry provided by the International Atomic Energy Agency (IAEA) [32].

**Table 1:** Details of the acquired commissioning and validation data.

**Data**	**Detector(s) & Geometry**	**Details**	**Agreement (Validation & End-to-End)**
Percent depth dose curves*	CC13, PTW 60019 microDiamond	Jaw FS = 4 × 4 cm – 40 × 40 cm	
Jaw CAX profiles*	IBA SFD, IBA CC13	Jaw FS = 1, 2, 3, 4, 6, 10, 20, 30, 40 cm	
		Depth = dmax, 5, 10, 20, 30 cm, SSD = 100 cm	
Dosimetric leaf gap*	PTW N30006 farmer chamber	Jaw FS = 10 × 10 cm	
		MLC gaps = 0.1, 0.5, 1, 2, 5, and 10 cm	
		Leaf speed = 20 cm/min	
		Measurement geometry modeled in Monte Carlo	
Point dose spot checks: Jaw fields	IBA CC13	Jaw FS = 5 × 5, 10 × 10, 30 × 30, 4 × 40, 40 × 4 cm. d = 5, 10, 20, 30 cm. CAX and off-axis. SSD = 90 cm	−0.57% ± 0.66% [−1.72%, 1.04%]
Point dose spot checks: MLCs	IBA CC13	MLC FS = 15 cm diameter. D = 5, 10, 20, 30 cm. CAX. SSD = 90 cm.	−0.40% ± 0.76% [−1.06%, 0.54%]
MLC central axis output factors*	IBA SFD	Multiple Jaw Settings. MLC FS = 0.5, 1, 2, 3, 4, 6, 10 cm. d = 10 cm. SSD = 90 cm	SFD: −0.83 ± 0.93 [−2.20%, 0.20%]
	PTW 60019 microDiamond		Diamond: −0.07% ± 0.53% [−1.01%, 1.39%]
	CC01 chamber		CC01: −0.26% ± 0.33% [−1.10%, 0.20%]
	SNC Edge		Edge: −0.31% ± 0.61% [−1.37%, 0.78%]
			Average: −0.45% ± 0.52% [−1.57%, 0.20%]
MLC central axis small field profile*	IBA SFD	MLC FS = 1 cm, d = 5 cm, SSD = 95 cm.	Inline ΔFWHM = 0.03 cm (2.7%), Crossline ΔFWHM = 0.02 cm (2.3%)
MLC off axis output factors*	IBA SFD	MLC FS = 0.5 cm, 1 cm, 2 cm, d = 10 cm, SSD = 90 cm.	SFD: −2.52% ± 1.12% [−5.06%, −0.23%]
	PTV 60019 microDiamond	Off axis distance from CAX = 2–10 cm inline/crossline.	Diamond: 0.00% ± 1.23% [−2.49%, 2.31%]
	IBA CC01 chamber		CC_01_: −1.65% ± 0.96% [−4.13%, −0.23%]
	SNC Edge		Edge: −0.56% ± 1.22% [−3.15%, 1.37%]Average: −1.32% ± 1.03% [−3.54%, 0.64%]
MLC off axis profiles*	IBA SFD	MLC FS = 1 cm, d = 5 cm, SSD = 95 cm.Offset by ± 5 cm and ± 9 cm inline and crossline.	ΔFWHM at 5 cm off axis: 0.02 cm (1.9%) ± 0.05 cm (5.0%) RMS = 0.05 cm (4.9%)ΔFWHM at 9 cm off axis: −0.01 cm (1.0%) ± 0.02 cm (2.5%) RMS = 0.02 cm (2.5%)
Single target DCA	SRS mapCHECK in StereoPHAN	PTV diameter = 0.5, 1, 2, 4 cm. PTV to MLC margin = 2 mm.	Isocenter dose per plan: 1.4%, 0.5%, 0.1%, and −0.5% for 0.5, 1, 2, & 4 cm targets, respectively.Gamma index (3%, 1 mm) = 100% for all deliveries.
		Jaw size = 1.6 × 1.6, 2.0 × 2.0, 2.5 × 2.5, 4.6 × 4.6 cm.PTV location = CAX. Detector centered on target.	
single isocenter VMAT SRS	SRS mapCHECK in StereoPHAN	21 targets from 10 clinical cases. PTV volume = 0.04–2.64cc. # targets = 3–7. Dist to iso = 0.7–8.3 cm. Detector centered on target. True composite delivery.	Gamma index (2%, 1 mm, 10% threshold) = 99.2% ± 2.1% [91.2%, 100%]High dose region (>80% Dmax): ΔDmean = 0.2% ± 1.1% [−1.5% 2.0%]
single isocenter VMAT SRS	OSLds & Exradin® W1 scintillator Detectors in STEEV phantom	6 targets. Diameter = 0.4–2.0 cm. Dist to iso = 1.2–7.9 cm.	Dose difference = −1.9% ± 2.2% [−5.0 1.1]
		OSLds centered in each target. 0.4 cm target measured using W1 scintillator.	OSLds only = −1.3% ± 1.8% [−3.8 1.1]. Scintillator only (0.4 cm target) = −5.0%.

*Data utilized in beam modeling process. CAX: Central Axis, FS: Field Size, SSD: Source to Surface Distance, MLCs: Multi-Leaf Collimators, d: depth, FWHM: Full Width Half Maximum, RMS: Root Mean Square, PTV: Planning Target Volume, SFD: Stereotactic Field Diode, OSLds: Optically Stimulated Luminescence dosimeters, DCA: Dynamic Conformal Arc, VMAT: Volumetric Modulated Arc Therapy, SRS: Stereotactic Radiosurgery.

#### 2.2.3 End-to-end testing

After beam data acquisition, modeling, & validation, a series of end-to-end tests comparing the SciMoCa Monte Carlo calculation to measurements with (1) the SRS MapCHECK diode array placed within the StereoPHAN phantom (Sun Nuclear Corporation, Melbourne FL), (2) Optically Stimulated Luminescence dosimeters (OSLd, Landauer, Glenwood, IL) and (3) a scintillation detector placed within an anthropomorphic Stereotactic End-to-End Verification (STEEV) phantom (CIRS, Norfolk, VA). Details for each of the end-to-end tests are summarized in Table 1.

The SRS MapCHECK measurements included simple dynamic conformal arc (DCA) treatment plans, and clinical single isocenter VMAT plans. The DCA plans were prepared for a spherical PTV centered on the detector plane of the SRS MapCHECK; plans were prepared with PTV diameters ranging from 0.5 to 4 cm. The single isocenter VMAT measurements were taken from clinical treatment plans prepared on patient geometry. The planning technique for these plans has been described in detail previously [2], [4], but consists of 3–6 non-coplanar VMAT arcs delivering 17.8 ± 2.25Gy [13Gy, 20Gy] prescribed to the 100% isodose line and normalized for adequate target coverage. A total of 21 PTVs were selected from 10 patient plans (half of all PTVs treated among the 10 cases) so as to have a variety of volumes, distances from isocenter, and number of PTVs per plan. In some cases, the detector plane included multiple PTVs, so that the total number of plan deliveries was 17 (to verify a total of 21 targets). The single isocenter VMAT plans were recalculated using the Monte Carlo SciMoCa beam model on the SRS MapCHECK / StereoPHAN geometry with selected target(s) positioned on the SRS MapCHECK detector plane. The original couch angles were kept for the phantom delivery so as to be able to verify a high dose region for the selected target (true composite delivery). Per the vendor’s instructions, the dose calculation (including for detector calibration) was carried out using a density override of 1.2g/cc (282HU) for the entire phantom; within SciMoCa this was accomplished by assigning the material of the phantom to be Polymethyl Methacrylate (PMMA or acrylic).

**Figure 1:**
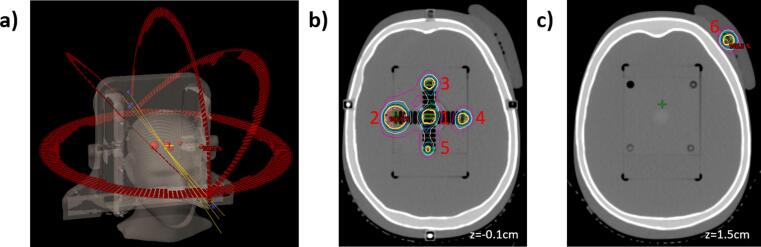
Plan and target geometry within anthropomorphic STEEV phantom for end-to-end verification test. Location of each target is annotated by numbers 1–6.

The STEEV phantom is an anthropomorphic phantom that includes realistic tissue and bone densities, and thus serves as a full end-to-end test in realistic geometry and includes inhomogeneity corrections. End-to-end measurements consisted of point dose measurements for single isocenter VMAT SRS with six PTVs, each with an OSLd placed within the PTV; a range of PTV volumes (0.1–4.1cc) and distances from isocenter (1.2–7.9 cm) were included to incorporate various clinical scenarios. The location of targets within the STEEV phantom along with arc geometry is illustrated in Fig. 1.

Each PTV was delivered 4Gy to the 100% isodose line, with 3 measurements carried out and averaged to improve statistics; dosimeter signal from imaging was measured separately and subtracted to determine measured dose. For each irradiation, the dosimeter within the OSLd casing was contoured in the TPS to get the exact location. For the smallest PTV (target #5 in Fig. 1, 0.4 cm diameter, 0.1cc), the active area of the OSLd was not small enough to obtain an accurate reading, so for this volume the measurement was made using a scintillator detector (Exradin® W1, Standard Imaging, Middleton WI), with the isocenter shifted so as to place the smallest PTV at the active area of the detector (with dose being recalculated with the shift applied).

### 2.3 Clinical implementation & action criteria

We quantified the agreement between dose calculated on patient anatomy using the Monte Carlo independent dose calculation algorithm and the dose calculation algorithm used in the TPS. This analysis was used to assess various potential action criteria, and to compare the results with the results from a pre-treatment QA performed using the SRS MapCHECK in StereoPHAN geometry. For the comparison, we utilized the same 10 patient cases from the end-to-end SRS MapCHECK measurement described above and in Table 1 using 2%/1 mm gamma criteria and a 10% low dose threshold. However, in this case we compare dose calculated on patient anatomy using the original AAA TPS calculation and Monte Carlo, and this comparison was made for all targets. For the subset of targets for which an SRS MapCHECK measurement was made (21 targets in 17 deliveries), the agreement calculated within patient anatomy was compared to the agreement of TPS AAA calculation on StereoPHAN geometry with the SRS MapCHECK measurement for several dosimetric indices.

To the extent possible, procedures and action criteria for the Monte Carlo independent dose calculation software were guided by the AAPM TG-219 report [24]. TG-219 provides recommendations for independent point dose calculation action levels depending on the clinical situation of a point dose calculation (homogeneous vs. heterogeneous, single beam vs. composite, high vs. low dose, high vs. low gradient), but does not provide DVH based action criteria for anatomical structures. TG-219 also does not provide specific recommendations regarding radiosurgery. Thus, for this study we quantified the dosimetric agreement of the independent calculation with the original TPS for DVH based metrics for structures relevant to radiosurgery. We then assessed the implications for pass/fail for various action levels, including any recommendations from TG-219. Specifically, we compare agreement for our DVH based metrics to the TG-219 recommended action levels of ±5% for a dose calculation in heterogeneous medium in a high dose / low gradient region, and ±7% for a dose calculation in heterogeneous medium in a low dose or high gradient region. We also calculated custom action levels for each dose metric following the guidelines outlined in the TG-218 report [25].

DVH based metrics evaluated for each individual radiosurgery target and for the combined target volume included the mean dose (D_mean_[Gy]), minimum dose received by the hottest 99% of volume (D_99%_[Gy]), hottest 95% of volume (D_95%_[Gy]), and hottest 1% of volume (D_1%_[Gy]). In addition, brain sub volume (brain minus PTV) receiving greater than 60% of the prescription dose (V_60%_[cc]), which corresponds to the V_12Gy_[cc] for a 20Gy prescription, was also evaluated.

The independent dose calculation software also includes an option for 3D Gamma Index (γ) comparison [33] between the TPS and Monte Carlo dose calculation. For Gamma Index Analysis of independent dose calculations, TG-219 [24] suggests following the recommendations of TG-218 [25]; this includes a universal criteria of 3%, 2 mm with an action limit of 90% passing voxels. Because this is not specific to SRS, we further modified it for our analysis and calculated fraction of voxels with Gamma ≤1 using a spatial criterion of 1 mm and dose criteria of 1–3%. The minimum dose threshold for this analysis was initially set to 15%, however higher thresholds were also evaluated in order to exclude low dose areas and focus on the more critical dose volumes (per TG-218 recommendation). Higher thresholds included 55% (so as to still include the V_60%_ / V_12Gy_ in the analysis), and 80% to focus exclusively on the high dose regions. Global normalization was used. Per TG-219 / TG-218 recommendations, custom action limits were also calculated from the measured Gamma Index values.

## 3 Results

### 3.1 Beam data acquisition, modeling, & validation

Agreement between the SciMoCa beam model and measurements made during the validation stage is summarized in the rightmost column of Table 1. A full report of the agreement between the Monte Carlo beam model and all the validation measurements from Table 1 is provided as a Supplementary data file.

### 3.2 End-to-end testing

**Figure 2:**
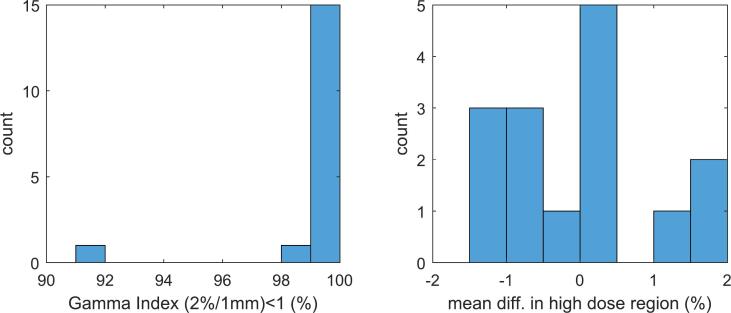
Comparative analysis between SciMoCa calculation and measurement with SRS MapCHECK in StereoPHAN phantom geometry for 21 PTVs (17 deliveries) taken from clinical SIMT SRS cases.

Key results from the end-to-end testing (SciMoCa vs. measurement) are summarized in the rightmost column of Table 1. For the DCA plans measured using the SRS MapCHECK in the StereoPHAN, the dose at isocenter agreed to within 1.4% for the smallest target, and within ±0.5% for the remaining plans. Using a Gamma Index analysis criteria of 3%/1 mm (absolute comparison, threshold = 10%), the percent of detectors with Gamma <1 was 100% for all dynamic conformal arc deliveries. Comparison between calculation and measurement for the single isocenter VMAT deliveries to the SRS MapCHECK illustrated in Fig. 2.

For these cases, the high dose region (defined as detectors with signal >80% of the maximum dose) agreed to within ±2% for all cases. Using a Gamma Index analysis criteria of 2%/1 mm (absolute comparison, threshold = 10%), the percentage of detectors with Gamma <1 was 99.2% on average and, except for one outlier at 91.2%, had a minimum of 98.6%. Further analysis of the outlier case showed a minor dose discrepancy outside of the high dose volume of interest; and when the threshold for the analysis was raised from 10% to 25%, the percent of detectors with Gamma Index <1 increased from 91.2% to 100%.

For the end-to-end tests using the anthropomorphic STEEV phantom, the mean difference between OSLd measurement and the Monte Carlo calculation was −1.3% ± 1.8% (per target differences of −0.8%, −1.5%, −3.8%, −1.4%, and −1.1%), while the difference for the smallest target measured using the scintillator detector was −5.0%. No trend was observed between dose differences and size of target or distance from isocenter.

### 3.3 Action criteria for independent dose calculation

**Figure 3:**
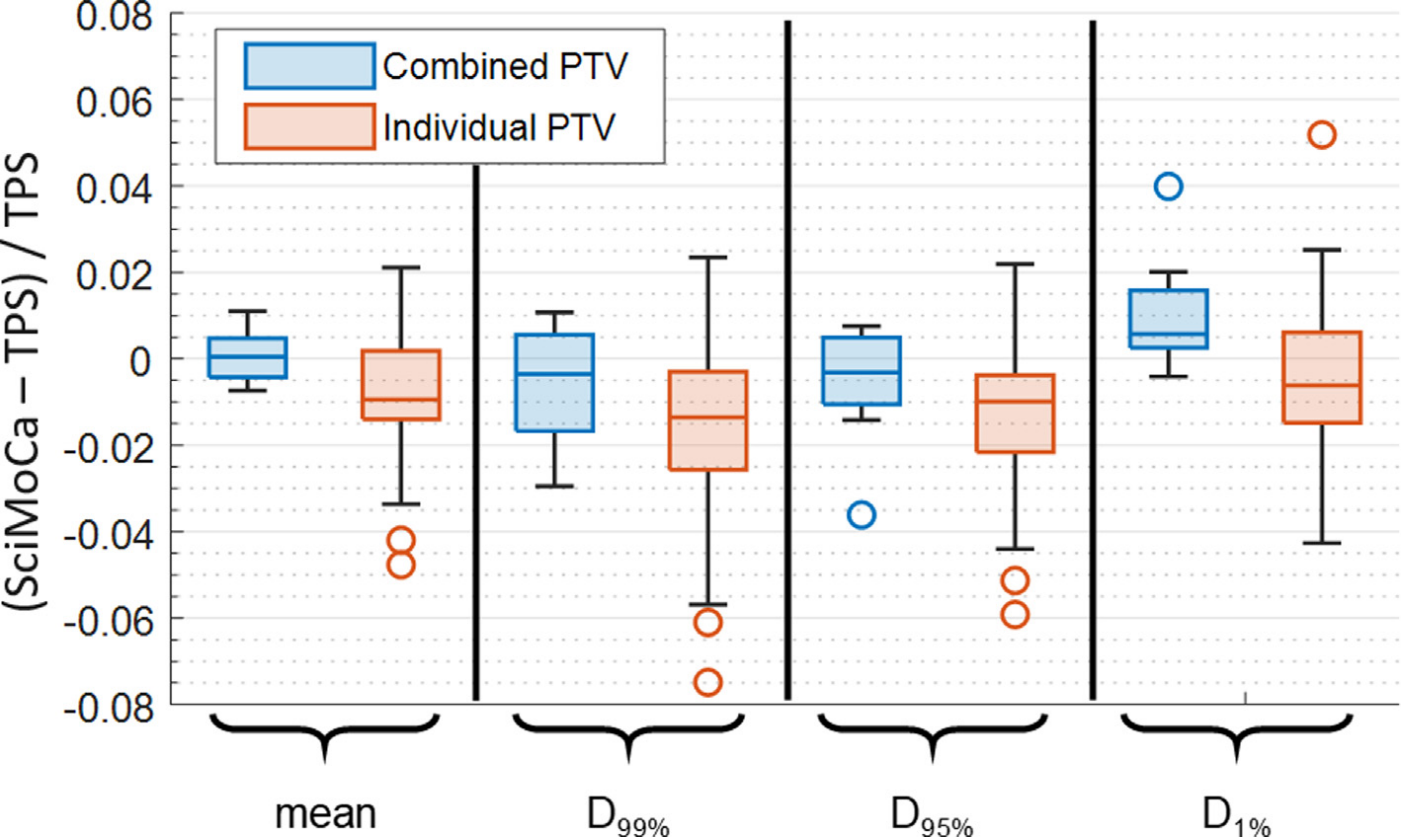
Difference between PTV dose metrics calculated on patient geometry using SciMoCa Monte Carlo and original TPS.

The difference between DVH based dose metrics calculated on the patient geometry using the TPS and Monte-Carlo for the individual and combined PTVs is illustrated in Fig. 3 and summarized in Table 2.

**Table 2:** Agreement between TPS and SciMoCa for the indicated dosimetric indices and patient volumes.

**Volume of interest**	**Comparative measure**	**Dose difference**	**TG-218 action limit**
combined PTV	mean dose	0.1% ± 0.5% [−0.7% 1.1%]	±1.6%
combined PTV	D_99%_	−0.5% ± 1.3% [−2.9% 1.1%]	±5.1%
combined PTV	D_95%_	−0.5% ± 1.3% [−3.6% 0.8%]	±4.3%
combined PTV	D_1%_	1.0% ± 1.3% [−0.4% 4.0%]	±4.2%
individual PTV	mean dose	−0.8% ± 1.5% [−4.8% 2.1%]	±5.2%
individual PTV	D_99%_	−1.6% ± 2.0% [−7.5% 2.3%]	±8.3%
individual PTV	D_95%_	−1.3% ± 1.7% [−5.9% 2.2%]	±6.4%
individual PTV	D_1%_	−0.5% ± 1.8% [−4.3% 5.2%]	±6.3%
plan	Gamma (3%, 1 mm, 15% threshold)	99.9% ± 0.2% [99.5% 100%]	98.8%
plan	Gamma (2%, 1 mm, 15% threshold)	99.8% ± 0.3% [99.2% 100%]	97.5%
plan	Gamma (1%, 1 mm, 15% threshold)	99.1% ± 0.7% [99.1% 99.9%]	93.3%
plan	Gamma (3%, 1 mm, 80% threshold)	99.5% ± 1.4% [95.5% 100%]	91.0%
plan	Gamma (2%, 1 mm, 80% threshold)	98.7% ± 2.5% [92.2% 100%]	83.0%
plan	Gamma (1%, 1 mm, 80% threshold)	96.7% ± 4.0% [87.4% 100%]	69.0%

A larger variation of differences was observed for individual PTVs relative to the combined PTV, as would be expected. All dose metrics for the combined PTVs fell within the TG-219 [24] recommended action levels of ±5% and ±7%. For the individual PTVs, all mean dose values fell within the ±5% action limit, while 3/42 (7%), 1/42 (2%) and 1/42 (2%) fell outside a ±5% action limit for D_99%_, D_95%_, and D_1%_, respectively; of these, only one target fell outside a ±7% threshold for a single metric (D_99%_). Customized action levels were also calculated following the procedure outlined in TG-218 [25] and are also summarized in Table 2. For brain V_60%_[cc], the difference between TPS and Monte Carlo was small at −0.25cc ± 0.27cc [−0.62cc 0.12cc]; for all cases the difference in predicted risk of radionecrosis [34] is <0.5%.

The results of the Gamma Index comparison (TPS vs Monte Carlo dose calculated on patient anatomy) are illustrated in Fig.re 4 and summarized in Table 2.

**Figure 4:**
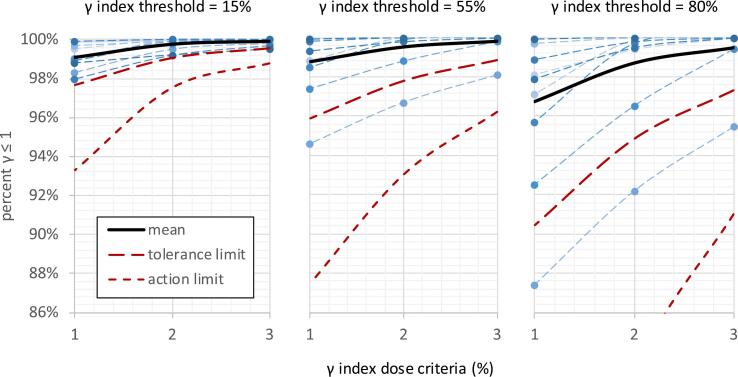
3D Gamma (γ) index comparison of Monte Carlo and original TPS dose calculation on patient anatomy for 10 multi-target SRS plans using various minimum dose thresholds and a DTA of 1 mm. Tolerance and action limits were calculated from the process outlined in TG-218 [25].

Agreement was very high for the Gamma analysis; for a 15% threshold all plans had >98% of voxels passing even with a 1%, 1 mm criteria. However, it is evident in Fig. 4 that the low dose threshold tends to mask dose discrepancies in the high dose region. When an 80% threshold is used (criteria = 1%, 1 mm), the agreement drops from 99.1% to 96.7% and differences between the various plans start to become manifest. For only one case was the passing fraction below the TG-218 universal action level of 90%, and this was only for stringent criteria of 1%/1 mm. Calculated tolerance and action limits were best aligned with the universal limits provided by TG-218 for analysis criteria of 3%/1 mm with an 80% threshold (tolerance limit = 97.3%, action limit = 91% compared to recommended universal limits of 95% and 90%, respectively).

In comparing the DVH based metrics with the Gamma Index analysis, the correlation between difference in near maximum combined PTV dose (D_1%_) and the Gamma Index passing fraction (3%, 1 mm, 80% threshold) was sufficiently large to reject a null hypothesis of no correlation. This was the case both for the combined PTV (R = -.80, p = 0.005) and the largest dose metric discrepancy of any individual PTV (R = -.64, p = 0.05). Correlation with other dose metrics did not reach the level of significance. Note that the correlation that was observed was driven primarily by a single plan for which the Gamma Index was lowest of all plans (95.5% at 3%/1 mm, 80% threshold) and the D_1%_ dose difference was largest of all plans (4.0% for PTV combined, 5.2% for individual PTV); when this case is omitted there is no substantial correlation. This is likely because this is the only plan for which the difference in D_1%_ was large enough that Gamma Index was also affected substantially. Whether this trend is verified when more cases with relatively larger dose discrepancies are accrued, remains to be seen.

### 3.4 Clinical application & integration with pre-treatment QA measurement

For the targets selected for pre-treatment QA measurement, the passing rate for the Gamma Index (2%, 1 mm, 10% threshold) comparison between SRS MapCHECK and original TPS dose calculation was 99.5% ± 0.9% [97.3% 100%]. Comparing the mean dose in the high dose region (80% dose threshold) in the detector plane with StereoPHAN geometry, the difference between calculated and measured was 0.1% ± 1.3% [−2.1% 2.3%]. In comparing the DVH based metrics (dose difference between TPS and Monte Carlo on patient anatomy) with the SRS MapCHECK pre-treatment QA measurement (mean dose difference between TPS and measurement for high dose region, a.k.a. 80% dose threshold on StereoPHAN geometry), the correlation was sufficiently large to reject a null hypothesis of no correlation for the PTV mean dose (R = 0.51, p = 0.038) and for D_1%_ (R = 0.57, p = 0.017). There was negligible correlation between Gamma Index results from the SRS MapCHECK QA and the DVH statistics comparison between TPS and Monte Carlo on patient anatomy. Notably, for the single target for which difference in D_99%_ calculated with TPS and Monte Carlo was outside a ±7% threshold, the SRS MapCHECK QA Gamma Index (2%/1 mm, 10% threshold) passing rate was 100%, and the dose difference within the high dose region (80% threshold) being minimal (0.7% ± 1.3%).

## 4 Discussion

In this study we commissioned and implemented a secondary Monte Carlo dose calculation algorithm capable of accurate volumetric dosimetry for radiosurgery of multifocal intracranial disease. The aim for this particular beam model is widespread availability via the software for use with single isocenter radiosurgery, which potential future users would be able to implement in their single isocenter multi-target radiosurgery practice. In this study, the beam was modeled and validated against an extensive set of measurements (Table 1), with the intent that future adopters of the independent dose calculation beam model for SRS may use this report as a benchmark comparison during the verification process.

The beam model used in this study benefits over the standard from the fine-tuning of the photon source diameter and the position calibration of leaves and jaws, for which output factors of small, MLC-delimited fields ≤20 × 20 mm^2^ as well as DLG measurements were available. These parameters could be determined to an accuracy of 0.05 mm and 0.03 mm, respectively. The inter-leaf air gap is determined from transmission measurements to an accuracy of 0.02 mm. These small tolerances already illustrate that small field output factor accuracy is ultimately limited by leaf positional uncertainty, lateral leaf play (especially for field positions far off-axis), and variations of the photon spot size. Evidence of these effects is present in the Supplementary material, where the cumulative effect on output factor can exceed 1% for fields ≤10 × 10 mm^2^. Since a MC model is always an idealization, this level of dose uncertainty can only be reduced if the treatment machine is monitored by a very strict QA regime that may include tests for off-axis performance.

Guidelines for volumetric independent dose calculation software was recently published in the AAPM TG 219 report [24]). While TG-219 addressed sophisticated volumetric dose calculation algorithms, it only briefly touched on Monte Carlo based algorithms as they were not commercially available at the time of preparation of the report. As part of our experience we calculated action criteria for DVH based metrics (Table 2). Little guidance exists currently on action criteria for DVH based metrics from independent dose calculations; however, TG-219 does recommend action levels for point dose calculations: ±5% for a dose calculation in heterogeneous medium in a high dose / low gradient region, and ±7% for a dose calculation in heterogeneous medium in a low dose or high gradient regions [24]. The custom action levels calculated here are for the most part comparable to the TG-219 recommended action levels. For D_95%_ and D_1%_, action limits of roughly ±5% and ±7% align well for the combined PTV and individual PTVs, respectively, while more stringent action limit(s) may be appropriate for mean dose (roughly ±2% and ±5%, respectively).

The capability of independent dose calculation algorithms has evolved dramatically, highlighting the evolving roles of various patient specific quality assurance checks. Independent dose calculations have historically consisted of simplified dose calculations at a single point, and lacked sufficient accuracy to provide useful feedback for cases lacking electronic equilibrium as is the case for multifocal intracranial radiosurgery [35]. Thus, the large majority of the burden to verify plan quality has historically fallen to the pre-treatment measurement. However, with the advent of sophisticated, volumetric independent dose calculation algorithms, studies now show that independent recalculation outperforms traditional measurement‐based IMRT QA methods in detecting unacceptable plans [36], [37], [38]. However, conventional measurement-based techniques are still needed to potentially catch errors associated with plan delivery. Ongoing innovations may further minimize the role of a pre-treatment measurement, such as online adaptive radiotherapy techniques (which are incompatible with a pre-treatment measurement), and development of pretreatment measurement substitutes such as artificial intelligence models to predict pre-treatment measurement outcomes [39] or predicted DVH at delivery based on predicted plan specific linear accelerator performance [40]. With these trends, a rigorous independent dose calculation, as well as clinical guidelines on standard practice and action limits for DVH metrics are increasing in importance.

## 5 Conclusion

In this work we reported the generation, validation and clinical benchmarking of a customized Monte Carlo beam model for independent dose calculation of single isocenter VMAT for treatment of multi-focal intracranial radiosurgery, which has widespread applicability via the commercial software. The results reported here may serve as a benchmark for clinics implementing the commercial independent Monte Carlo dose calculation for single-isocenter radiosurgery for multifocal disease. We reported agreement between Monte Carlo and measured beam data (central axis and off axis), as well as end-to-end measurements. Regarding DVH based metrics for comparison between TPS and Monte Carlo dose calculated on patient anatomy, custom action levels were generally in agreement with TG-219 recommended action limits for point dose. For 3D Gamma Index comparison of TPS vs. Monte Carlo on patient anatomy, a low minimum dose threshold of 15% tended to mask dose differences in the high dose volume, while a value of 80% was better able to differentiate relevant dose differences; criteria of 3%/1 mm resulted in action levels similar to the TG-218 recommended universal criteria. Difference between TPS and Monte Carlo for individual target mean and D_1%_ was shown to be correlated with the difference between calculated and measured dose to the high dose region for a true composite delivery to the SRS MapCHECK.

Monte Carlo based independent secondary dose calculation has promise to enhance pre-treatment QA of single isocenter radiosurgery of multifocal intracranial disease by providing comprehensive dosimetric analysis for all individual targets.

## Funding

Funded in part by a grant by Radialogica LLC.

## Declaration of Competing Interest

The authors declare the following financial interests/personal relationships which may be considered as potential competing interests: Drs. Adamson & Kirkpatrick report ownership of Clearsight RT LLC which is unrelated to this study. Dr. Adamson reports research funding from Radialogica LLC, which is a distributor of the SciMoCa independent dose calculation software. Dr. Alber is a founder of Scientific-RT, which manufactures the SciMoCa Monte Carlo independent dose calculation algorithm.

## Appendix A Supplementary data

Supplementary data 1 Summary of commissioning measurements for the SciMoCa beam model.
