# Supplementary material for: Generation, validation, and benchmarking of a commercial independent Monte Carlo calculation beam model for multi-target SRS
Source: Z Med Phys. 2023 Sep 14;35(3):248–58. doi: 10.1016/j.zemedi.2023.08.004 (PMC12664461; doi:10.1016/j.zemedi.2023.08.004)
Supplement: Supplementary data 1 — Summary of commissioning measurements for the SciMoCa beam model. [file mmc1.pdf]

Customized Monte Carlo Independent Calculation Beam Model for Multi-Target SRS

Supplementary Data File: Agreement with measurement for SciMoCa Monte Carlo Beam Model Customized for Single Target SRS

All measurements and calculations were performed for a 6XFFF photon beam from a Varian STX linear accelerator with HD-MLC. Dose calculation utilized the SciMoCa HiTune beam model, which is a 6XFFF beam model within SciMoCa that has been customized for dose calculation of single isocenter SRS.

1. Point Dose Spot Checks: Jaw Fields

Point dose spot check measurements were verified for fields defined by jaws with calculation for the following conditions:

- Detector: CC13 chamber in water
- Jaw FS = 5x5, 10x10, 30x30, 4x40, 40x4 cm^2
- Depth = 5, 10, 20, 30 cm
- SSD = 90 cm

					STX 6XFFF
Open/Wedge	fieldsize Y x X, SSD	depth (cm)	inline (cm)	crossline (cm)	CC13 vs SciMoCa (% difference)
Open/SSD=100	10x10, 100SSD	10	0	0	-0.11%
	10x10, 90SSD	5	0	0	-0.86%
	10x10, 90SSD	10	0	0	-0.29%
	10x10, 90SSD	20	0	0	0.37%
	10x10, 90SSD	30	0	0	0.36%
	5x5, 90SSD	5	0	0	-0.28%
	5x5, 90SSD	10	0	0	-0.61%
	5x5, 90SSD	20	0	0	-0.45%
	5x5, 90SSD	30	0	0	-0.16%
	4x40, 90SSD	5	0	0	-0.45%
	4x40, 90SSD	10	0	0	-0.54%
	4x40, 90SSD	20	0	0	0.11%
	4x40, 90SSD	30	0	0	1.04%
	20x20, 90SSD	10	-7	0	-0.60%
	20x20, 90SSD	10	-3	0	-0.62%
	20x20, 90SSD	10	0	0	-1.56%
	20x20, 90SSD	10	3	0	-0.91%
	20x20, 90SSD	10	7	0	-1.57%
	20x20, 90SSD	10	0	-7	-1.08%
	20x20, 90SSD	10	0	-3	-0.83%
	20x20, 90SSD	10	0	3	-1.13%
	20x20, 90SSD	10	0	7	-1.72%
	20x20, 90SSD	20	-7	0	0.90%
	20x20, 90SSD	20	-3	0	-1.08%
	20x20, 90SSD	20	0	0	-0.49%
	20x20, 90SSD	20	3	0	-0.80%
	20x20, 90SSD	20	7	0	-0.51%
	20x20, 90SSD	20	0	-7	-0.68%
	20x20, 90SSD	20	0	-3	-1.21%
	20x20, 90SSD	20	0	3	-0.51%
	20x20, 90SSD	20	0	7	-1.29%
	**average (all)**				**-0.57%**
	**standard deviation (all)**				**0.66%**
	**max (all)**				**1.04%**
	**min (all)**				**-1.72%**

Results Summary:

- Difference between CC13 chamber measurement and Monte Carlo: -0.57% ± 0.66% [-1.72%, 1.04%]

2. Point Dose Spot Checks: MLC Fields

Point dose spot check measurements were verified for fields defined by MLCs with calculation for the following conditions:

- Detector: CC13 chamber in water
- MLC FS = 15 cm diameter.
- Depth = 5, 10, 20, 30 cm
- SSD = 90 cm

fieldsize Y x X, SSD	depth (cm)	Ion chamber - Monte Carlo (%)
25x25, 15cm dia MLC, 90SSD	5	-0.97%
25x25, 15cm dia MLC, 90SSD	10	-1.06%
25x25, 15cm dia MLC, 90SSD	20	-0.11%
25x25, 15cm dia MLC, 90SSD	30	0.54%
	average:	-0.40%
	stdev:	0.76%
	max	0.54%
	min	-1.06%

Results Summary:

- Difference between IBA CC13 chamber measurement and Monte Carlo: -0.40% ± 0.76% [-1.06%, 0.54%]

3. Depth-Dose-Curves: Jaw Fields

Depth-Dose-Curve measurements were verified for fields defined by jaws with calculation for the following conditions:

- Detector: CC13 chamber in water
- Jaw FS = 3x3, 4x4, 10x10, 20x20 cm^2.
- Depth = 0-25 cm
- SSD = 90 cm
- Monte Carlo uncertainty: 0.25% for 10x10 cm^2, 0.5% else

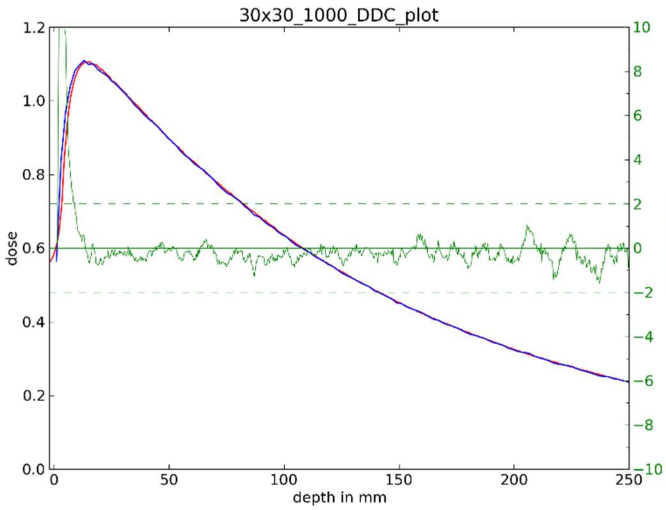

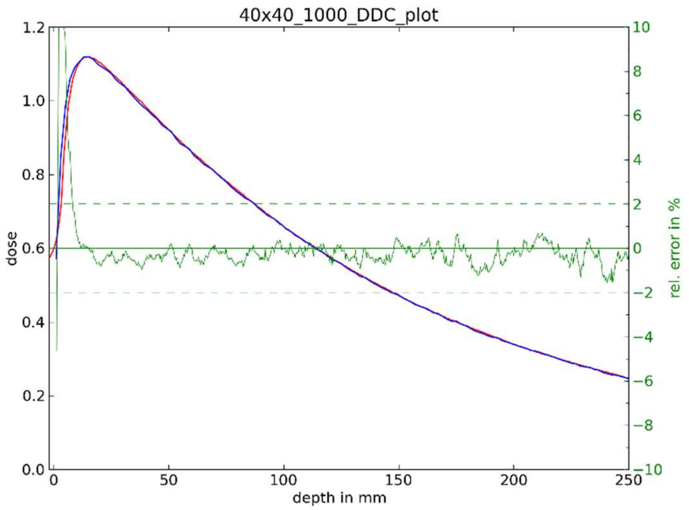

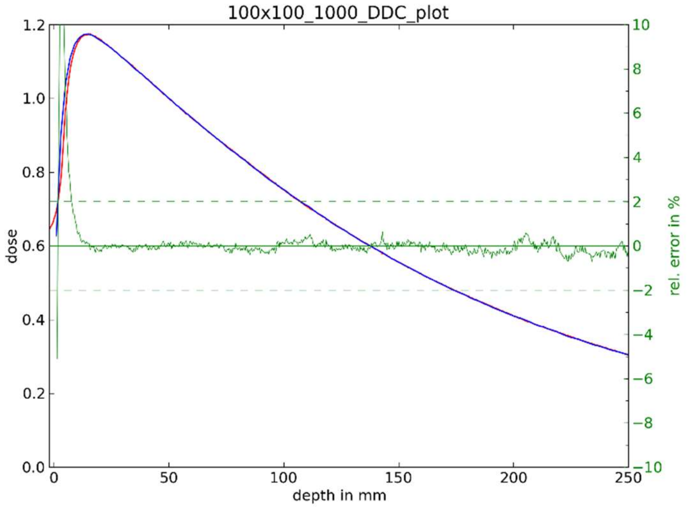

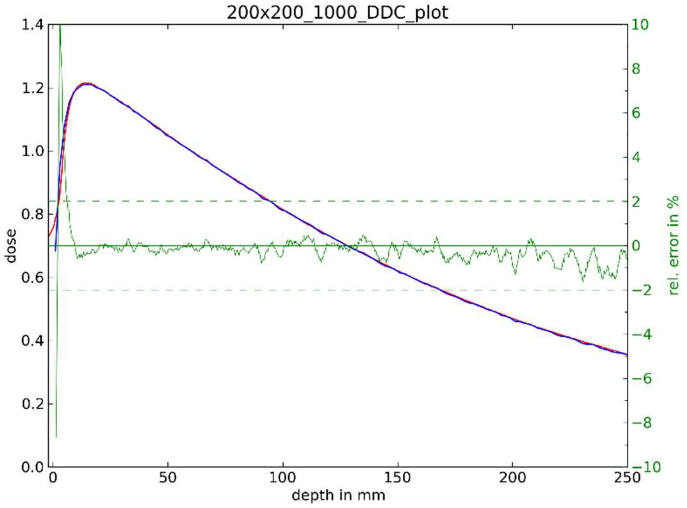

Results Summary:

- Absolute difference between simulations (blue) and measurements (red) shown as green curve typically within Monte Carlo uncertainty at depths from 2 to 20 cm for all fields, showing correct primary photon spectrum.

4. MLC Central Axis Output Factors

Small field output factors were measured using various detectors, including the small field correction factors provided by the IAEA TRS 483 report. These measurements were compared with the factors calculated using SciMoCa.

- Depth = 10 cm
- SSD = 90 cm
- Detectors (in water): IBA Stereotactic Field Diose (SFD), PTW 60019 Micro-Diamond, IBA CC01 microchamber, SunNuclear Edge Detector

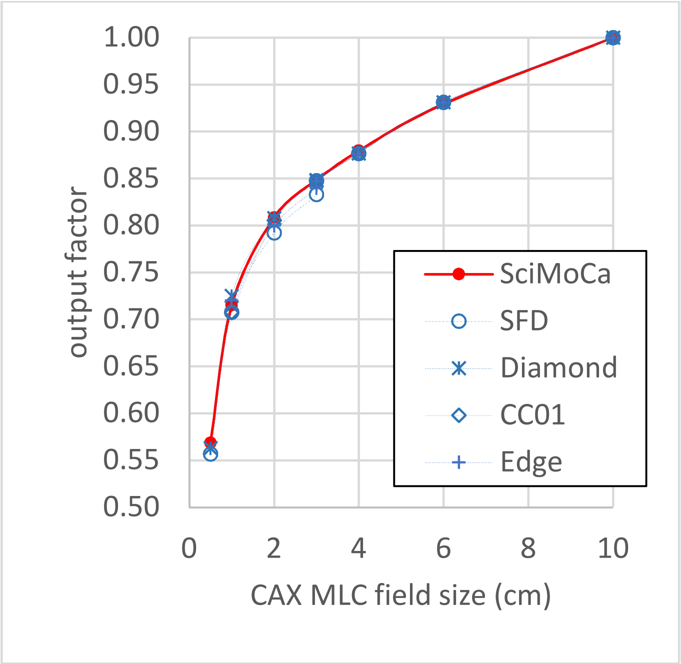

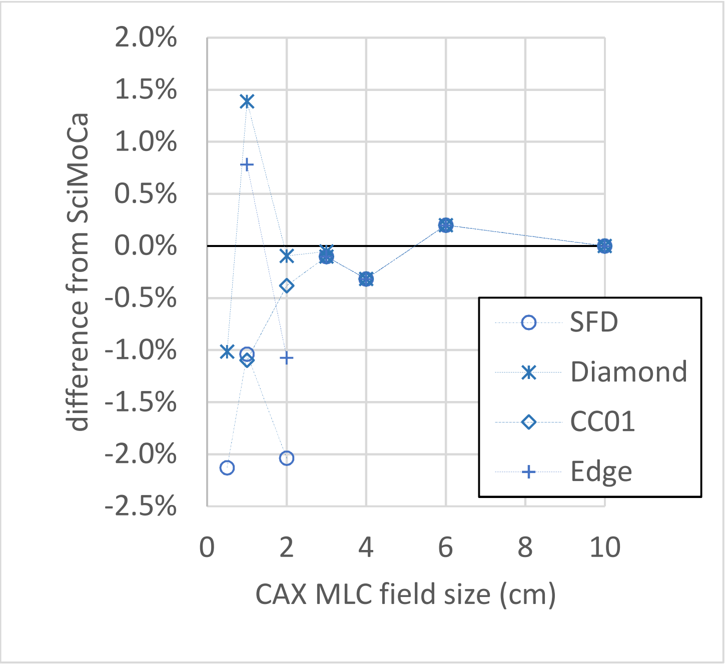

Difference between measured and calculated central axis output factors:

	SFD	Diamond	CC01	Edge	Average all detectors
Average	-0.9%	-0.1%	-0.3%	-0.4%	-0.4%
Standard deviation	0.9%	0.5%	0.3%	0.6%	0.5%
Min	-2.2%	-1.0%	-1.1%	-1.4%	-1.6%
Max	0.2%	1.4%	0.2%	0.8%	0.2%

Difference between measurement and SciMoCa Monte Carlo was <0.4% for MLC fields greater than 2x2cm^2. For a 1x1cm^2 MLC field size, difference between Monte Carlo calculation and measurement was -1.0%, 1.4%, -1.1%, and 0.8% for the SFD, Diamond, CC01, and Edge detectors respectively. For a 0.5x0.5 cm^2 MLC field size, difference between calculation and measurement was -2.1% and -1.0% for SFD and Diamond detectors, respectively (1x1 cm^2 was the smallest field size measured for CC01 and Edge).

5. MLC Central Axis Output Factors for Various Jaw Settings

Small field output factors were measured using the PTW 60019 Micro-Diamond detector including the small field correction factors provided by the IAEA TRS 483 report. MLC-collimated fields were measured with various jaw settings to investigate the influence of backscatter into the monitor chamber. These measurements were compared with the factors calculated using SciMoCa.

- Depth = 10 cm
- SSD = 90 cm
- Field sizes: 0.5x0.5 to 14x14 cm^2
- Monte Carlo uncertainty: 0.25%

Table of all measured output factors. Relative Difference in per cent between Monte Carlo and measurement (negative value: MC < measurement). Green cells: fields collimated by jaws.

		Jaw Field Size [mm]								
		8	12	22	32	42	60	80	**100**	140
MLC Field Size [mm]	5	-0.5	0.4	-0.2	0.1	0.0	0.1	0.1	0.0	-0.1
	10	-1.7	0.1	0.3	-0.2	0.3	0.1	0.2	0.2	0.1
	20		-0.2	0.1	-0.2	0.0	0.3	0.3	0.2	0.3
	30			0.0	-0.2	-0.2	-0.3	-0.1	-0.1	-0.2
	40				-0.1	0.0	-0.1	-0.3	-0.1	-0.3
	60					-0.3	-0.2	-0.1	0.1	0.2
	80							-0.1	-0.3	
	**100**								0.0	
	140									-0.2

Results Summary:

- Except for the 0.8x0.8 cm^2 jaw-collimated field, all output factor differences are within +-0.5%. The large deviation of the former is most likely due to jaw positioning errors in the range of tenths of mm.

6. MLC Central Axis Profiles

Small field profiles at central axis were measured using the IBA SFD in a water tank. These measurements were compared with the profiles calculated using the SciMoCa.

- Depth = 5 cm
- SSD = 95 cm
- Field size: 1x1 cm^2 with MLCs
- Detector (in water): IBA Stereotactic Field Diose (SFD)

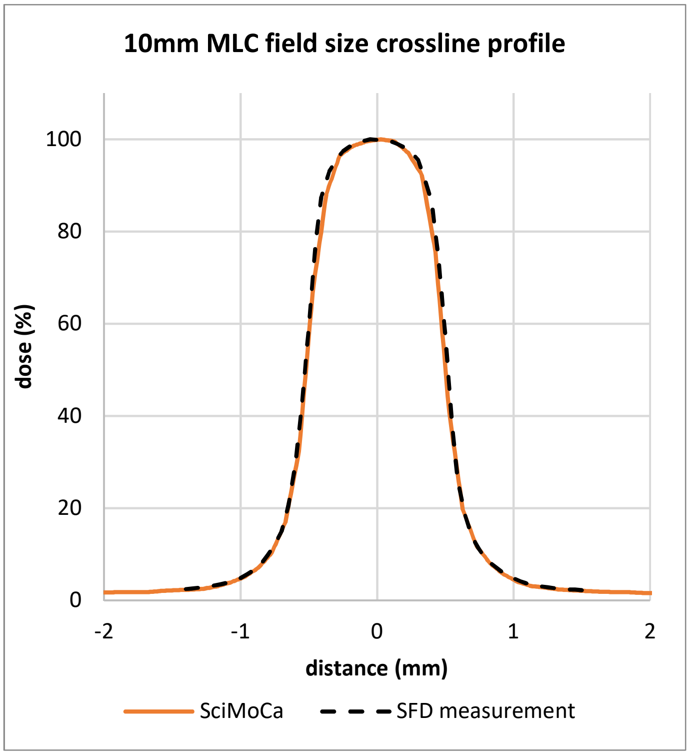

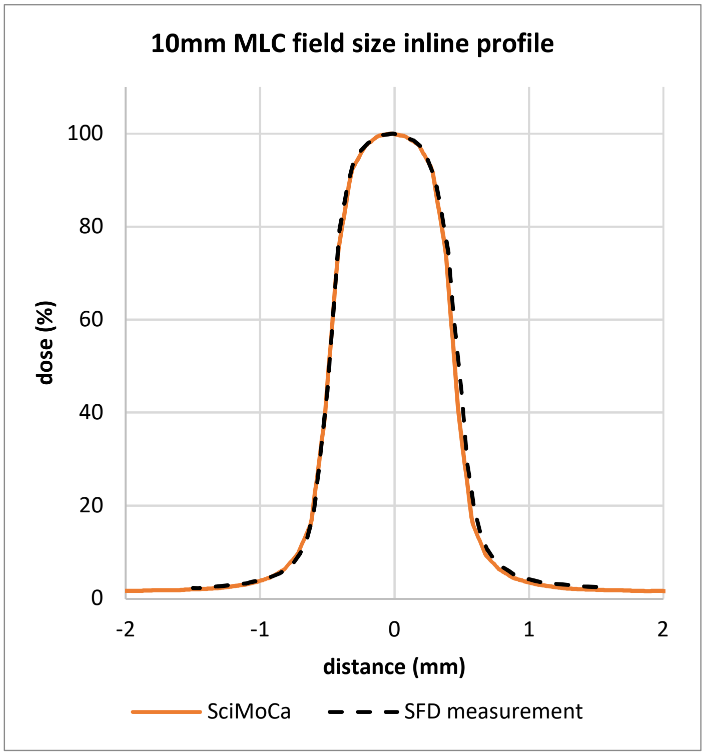

7. MLC Off Axis Output Factors

Small field output factors were measured at off axis locations using various detectors, including the small field correction factors provided by the IAEA TRS 483 report. These measurements were compared with the factors calculated using the SciMoCa

- Depth = 10 cm
- SSD = 90 cm
- MLC field sizes: 0.5x0.5 cm^2, 1x1 cm^2, 2x2 cm^2
- Distances off axis (along leaf motion axis): 2 cm, 3.5 cm, 6 cm, 8 cm, 10 cm
- Distances off axis (cross leaf axis): 2 cm, 3.5 cm, 5.5 cm, 7.5 cm, 9.5 cm
- Detectors (in water): IBA Stereotactic Field Diose (SFD), PTW 60019 Micro-Diamond, IBA CC01 microchamber, SunNuclear Edge Detector

Table of all measured output factors:

MLC (cm)	Jaw (cm)	Detector Pos. (cm)			SciMoCa	SFD		Diamond		CC01		EDGE	
		x	*y*	*z*	OF	*OF*	*% Diff*	*OF*	*% Diff*	*OF*	*% Diff*	*OF*	*% Diff*
10x10	10x10	0.00	0.00	10.00	1.0000	1.0000	0.000%	1.0000	0.000%	1.0000	0.000%	1.0000	0.000%
6x6	6x6	0.00	0.00	10.00	0.9293	0.9312	0.200%	0.9312	0.200%	0.9312	0.200%	0.9312	0.200%
4x4	4x4	0.00	0.00	10.00	0.8796	0.8768	-0.317%	0.8768	-0.317%	0.8768	-0.317%	0.8768	-0.317%
3x3	3x3	0.00	0.00	10.00	0.8487	0.8478	-0.103%	0.8478	-0.103%	0.8478	-0.103%	0.8478	-0.103%

2x2	2x2	0.00	0.00	10.00	0.8086	0.7921	-2.038%	0.8078	-0.097%	0.8055	-0.383%	0.7999	-1.073%
6x6	10x10	0.00	0.00	10.00	0.7151	0.7077	-1.038%	0.7250	1.387%	0.7072	-1.097%	0.7207	0.784%
4x4	10x10	0.00	0.00	10.00	0.5687	0.5566	-2.129%	0.5630	-1.014%				
3x3	10x10	0.00	0.00	10.00	0.9408	0.9384	-0.254%	0.9384	-0.254%	0.9384	-0.254%	0.9384	-0.254%
3x3	10x10	0.00	0.00	10.00	0.8940	0.8913	-0.302%	0.8913	-0.302%	0.8913	-0.302%	0.8913	-0.302%
2x2	10x10	0.00	0.00	10.00	0.8617	0.8611	-0.068%	0.8611	-0.068%	0.8611	-0.068%	0.8611	-0.068%
2x2	3x3	0.00	0.00	10.00	0.8148	0.7974	-2.141%	0.8142	-0.069%	0.8091	-0.700%	0.8076	-0.885%
2x2	3x3	2.00	0.00	10.00	0.7963	0.7779	-2.308%	0.7953	-0.127%	0.7897	-0.826%	0.7893	-0.884%
2x2	3x3	3.50	0.00	10.00	0.7661	0.7488	-2.261%	0.7645	-0.215%	0.7589	-0.942%	0.7580	-1.059%
2x2	3x3	0.00	2.00	10.00	0.7944	0.7779	-2.074%	0.7966	0.273%	0.7926	-0.227%	0.7905	-0.496%
2x2	3x3	0.00	3.50	10.00	0.7658	0.7495	-2.125%	0.7648	-0.134%	0.7603	-0.716%	0.7598	-0.785%
2x2	3x3	0.00	5.50	10.00	0.7156	0.6995	-2.256%	0.7134	-0.310%	0.7108	-0.667%	0.7076	-1.125%
2x2	3x3	0.00	7.50	10.00	0.6644	0.6479	-2.484%	0.6601	-0.653%	0.6563	-1.217%	0.6541	-1.547%
2x2	3x3	0.00	9.50	10.00	0.6150	0.5993	-2.549%	0.6096	-0.872%	0.6040	-1.797%	0.6039	-1.801%
2x2	10x10	6.00	0.00	10.00	0.7098	0.6905	-2.720%	0.7034	-0.898%	0.6972	-1.776%	0.6978	-1.684%
2x2	10x10	8.00	0.00	10.00	0.6575	0.6404	-2.597%	0.6507	-1.027%	0.6456	-1.817%	0.6459	-1.771%
2x2	10x10	10.00	0.00	10.00	0.6102	0.5918	-3.007%	0.6003	-1.619%	0.5953	-2.435%	0.5954	-2.423%
2x2	3x3	0.00	0.00	20.00	0.4066	0.3931	-3.327%	0.4063	-0.070%	0.4017	-1.211%	0.4015	-1.265%
2x2	3x3	2.20	0.00	20.00	0.3958	0.3841	-2.955%	0.3967	0.222%	0.3909	-1.233%	0.3919	-0.992%
2x2	3x3	3.85	0.00	20.00	0.3820	0.3692	-3.362%	0.3806	-0.361%	0.3737	-2.172%	0.3764	-1.461%
2x2	3x3	0.00	2.20	20.00	0.3966	0.3841	-3.151%	0.3970	0.101%	0.3945	-0.528%	0.3921	-1.131%
2x2	3x3	0.00	3.85	20.00	0.3823	0.3699	-3.242%	0.3806	-0.439%	0.3759	-1.686%	0.3770	-1.382%
2x2	3x3	0.00	6.05	20.00	0.3561	0.3438	-3.468%	0.3546	-0.420%	0.3522	-1.099%	0.3508	-1.494%
2x2	3x3	0.00	8.25	20.00	0.3300	0.3183	-3.532%	0.3276	-0.720%	0.3249	-1.537%	0.3234	-1.991%
2x2	3x3	0.00	10.45	20.00	0.3060	0.2914	-4.758%	0.3019	-1.331%	0.2970	-2.956%	0.2974	-2.815%
2x2	10x10	6.60	0.00	20.00	0.3532	0.3393	-3.945%	0.3498	-0.967%	0.3443	-2.521%	0.3458	-2.099%
2x2	10x10	8.80	0.00	20.00	0.3267	0.3139	-3.930%	0.3228	-1.192%	0.3149	-3.616%	0.3187	-2.436%
2x2	10x10	11.00	0.00	20.00	0.3030	0.2892	-4.555%	0.2965	-2.156%	0.2905	-4.126%	0.2935	-3.150%
1x1	3x3	0.00	0.00	10.00	0.7151	0.7077	-1.038%	0.7250	1.387%	0.7072	-1.097%	0.7207	0.784%
1x1	3x3	2.00	0.00	10.00	0.6974	0.6900	-1.071%	0.7079	1.498%	0.6886	-1.268%	0.7039	0.930%
1x1	3x3	3.50	0.00	10.00	0.6677	0.6627	-0.754%	0.6800	1.836%	0.6621	-0.850%	0.6753	1.131%
1x1	3x3	0.00	2.00	10.00	0.6955	0.6922	-0.483%	0.7104	2.139%	0.6922	-0.484%	0.7051	1.372%
1x1	3x3	0.00	3.50	10.00	0.6693	0.6656	-0.548%	0.6806	1.689%	0.6656	-0.549%	0.6761	1.013%
1x1	3x3	0.00	5.50	10.00	0.6271	0.6199	-1.148%	0.6343	1.148%	0.6212	-0.952%	0.6294	0.362%
1x1	3x3	0.00	7.50	10.00	0.5774	0.5750	-0.422%	0.5877	1.789%	0.5724	-0.869%	0.5824	0.859%
1x1	3x3	0.00	9.50	10.00	0.5342	0.5330	-0.231%	0.5418	1.419%	0.5308	-0.637%	0.5378	0.672%
1x1	10x10	6.00	0.00	10.00	0.6164	0.6104	-0.987%	0.6258	1.515%	0.6111	-0.863%	0.6202	0.611%
1x1	10x10	8.00	0.00	10.00	0.5721	0.5639	-1.434%	0.5782	1.067%	0.5659	-1.082%	0.5732	0.183%
1x1	10x10	10.00	0.00	10.00	0.5280	0.5219	-1.149%	0.5332	0.994%	0.5236	-0.824%	0.5289	0.183%
1x1	3x3	0.00	0.00	20.00	0.3551	0.3472	-2.221%	0.3604	1.509%	0.3472	-2.231%	0.3570	0.530%
1x1	3x3	2.20	0.00	20.00	0.3446	0.3384	-1.820%	0.3516	2.014%	0.3393	-1.552%	0.3487	1.180%
1x1	3x3	3.85	0.00	20.00	0.3329	0.3251	-2.335%	0.3376	1.431%	0.3228	-3.028%	0.3343	0.434%
1x1	3x3	0.00	2.20	20.00	0.3446	0.3406	-1.164%	0.3525	2.305%	0.3386	-1.746%	0.3493	1.363%
1x1	3x3	0.00	3.85	20.00	0.3348	0.3273	-2.239%	0.3379	0.939%	0.3264	-2.517%	0.3345	-0.091%
1x1	3x3	0.00	6.05	20.00	0.3112	0.3037	-2.414%	0.3148	1.149%	0.3041	-2.278%	0.3109	-0.106%
1x1	3x3	0.00	8.25	20.00	0.2860	0.2809	-1.783%	0.2901	1.438%	0.2783	-2.674%	0.2871	0.409%
1x1	3x3	0.00	10.45	20.00	0.2649	0.2587	-2.331%	0.2672	0.878%	0.2568	-3.068%	0.2640	-0.332%
1x1	10x10	6.60	0.00	20.00	0.3063	0.2985	-2.545%	0.3100	1.207%	0.2991	-2.361%	0.3068	0.142%
1x1	10x10	8.80	0.00	20.00	0.2834	0.2764	-2.463%	0.2859	0.894%	0.2762	-2.560%	0.2826	-0.272%
1x1	10x10	11.00	0.00	20.00	0.2636	0.2543	-3.509%	0.2628	-0.289%	0.2546	-3.388%	0.2601	-1.319%
0.5x0.5	3x3	0.00	0.00	10.00	0.5687	0.5566	-2.129%	0.5630	-1.014%				
0.5x0.5	3x3	2.00	0.00	10.00	0.5549	0.5418	-2.366%	0.5495	-0.972%				
0.5x0.5	3x3	3.50	0.00	10.00	0.5345	0.5205	-2.625%	0.5276	-1.292%				
0.5x0.5	3x3	0.00	2.00	10.00	0.5531	0.5418	-2.049%	0.5507	-0.427%				
0.5x0.5	3x3	0.00	3.50	10.00	0.5343	0.5212	-2.456%	0.5256	-1.632%				
0.5x0.5	10x10	6.00	0.00	10.00	0.4939	0.4801	-2.787%	0.4867	-1.454%				
0.5x0.5	10x10	8.00	0.00	10.00	0.4562	0.4440	-2.676%	0.4503	-1.298%				
0.5x0.5	10x10	10.00	0.00	10.00	0.4265	0.4122	-3.366%	0.4159	-2.485%				
0.5x0.5	3x3	0.00	0.00	20.00	0.2779	0.2677	-3.664%	0.2758	-0.735%				
0.5x0.5	3x3	2.20	0.00	20.00	0.2712	0.2599	-4.183%	0.2690	-0.824%				

0.5x0.5	3x3	3.85	0.00	20.00	0.2568	0.2507	-2.375%	0.2580	0.473%				
0.5x0.5	3x3	0.00	2.20	20.00	0.2717	0.2613	-3.835%	0.2698	-0.719%				
0.5x0.5	3x3	0.00	3.85	20.00	0.2577	0.2507	-2.713%	0.2583	0.246%				
0.5x0.5	10x10	6.60	0.00	20.00	0.2394	0.2309	-3.584%	0.2379	-0.661%				
0.5x0.5	10x10	8.80	0.00	20.00	0.2238	0.2125	-5.064%	0.2193	-2.019%				
0.5x0.5	10x10	11.00	0.00	20.00	0.2063	0.1969	-4.546%	0.2021	-2.027%				

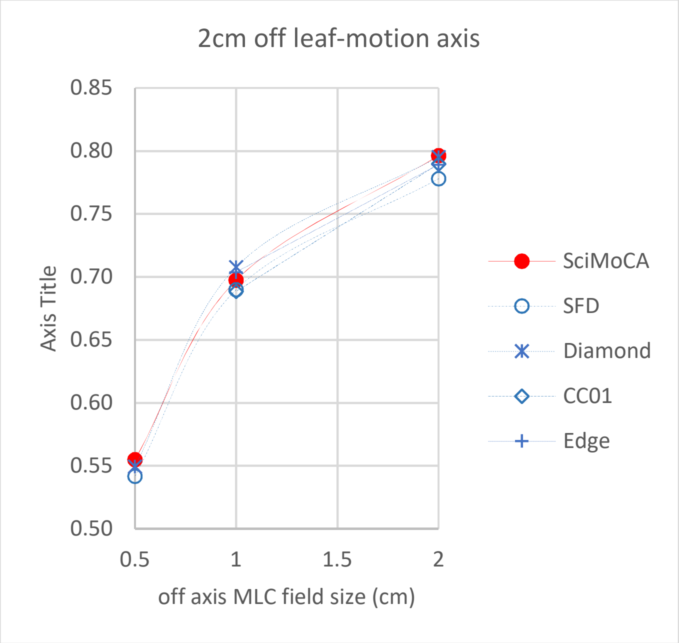

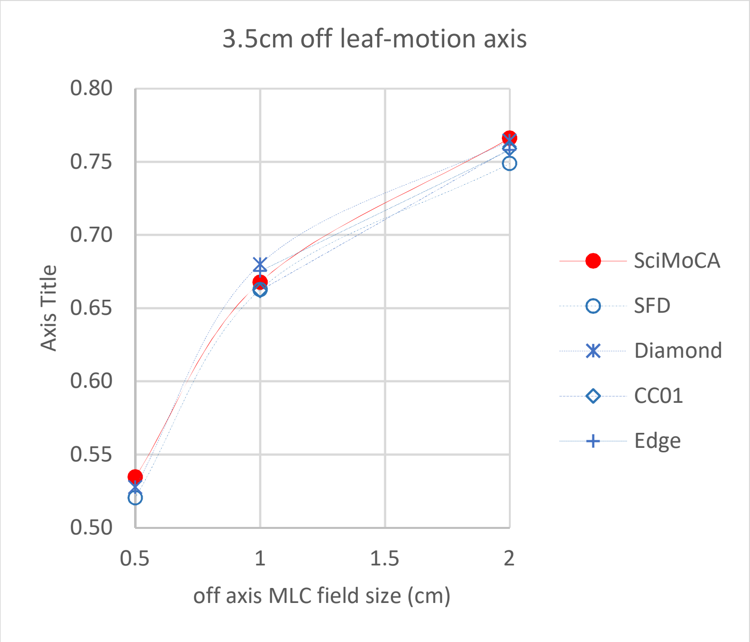

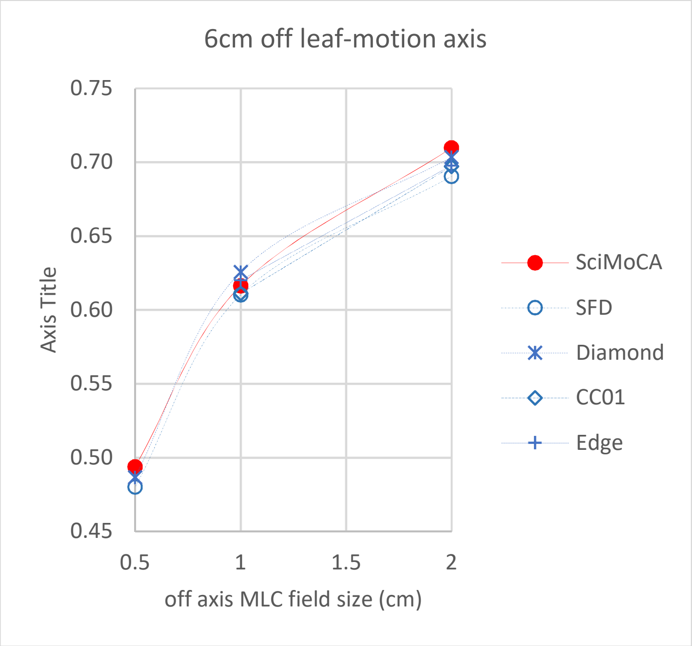

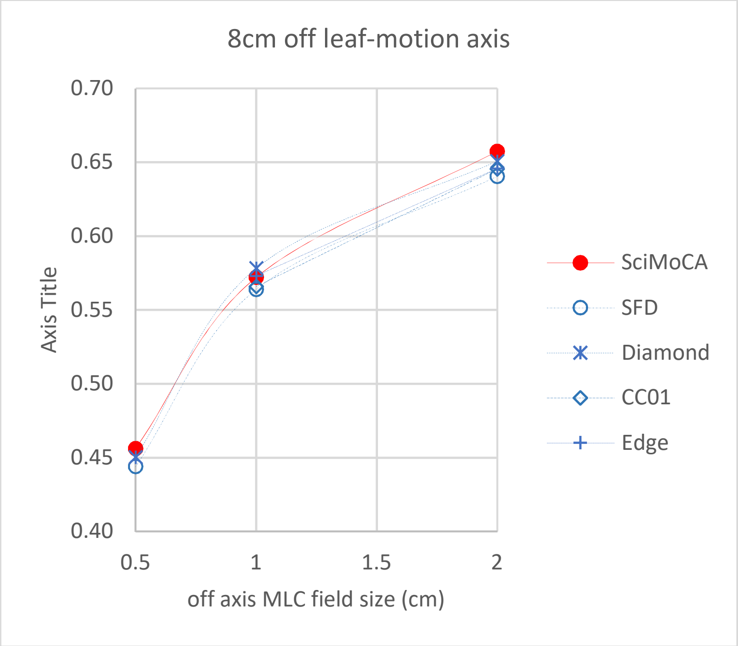

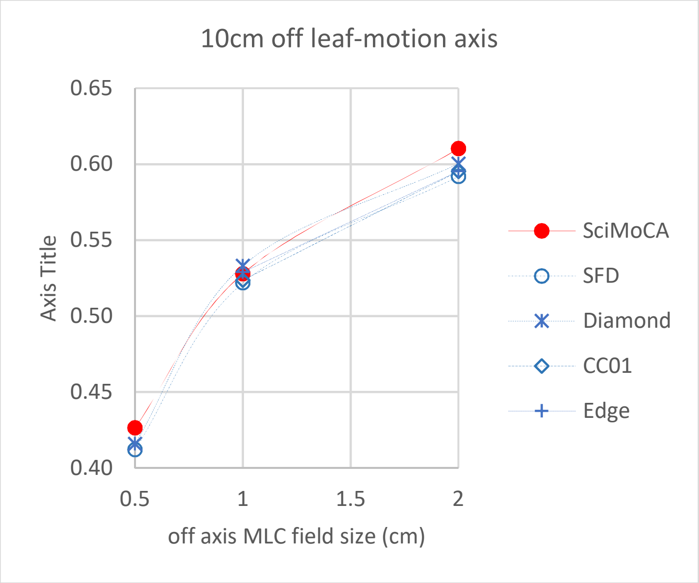
10cm off leaf-motion axis
Axis Title
0.65
0.60
0.55
0.50
0.45
0.40
0.5
1
1.5
2
off axis MLC field size (cm)
SciMoCA
SFD
Diamond
CC01
Edge

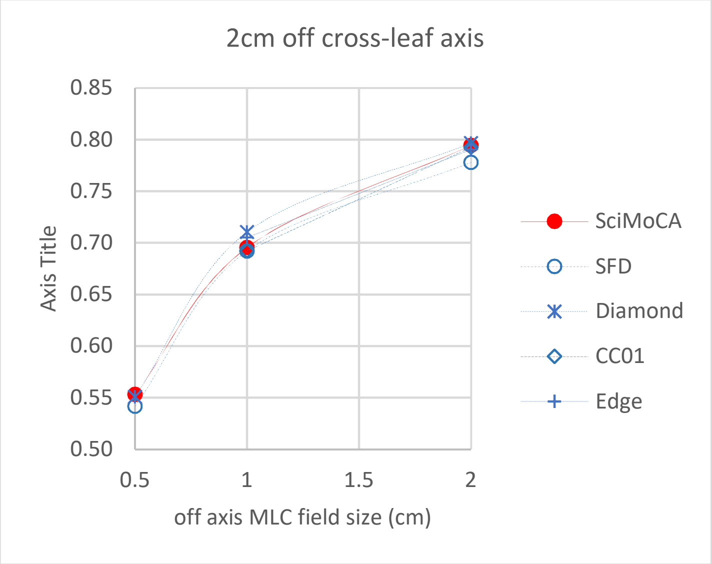
2cm off cross-leaf axis
Axis Title
0.85
0.80
0.75
0.70
0.65
0.60
0.55
0.50
0.5
1
1.5
2
off axis MLC field size (cm)
SciMoCA
SFD
Diamond
CC01
Edge

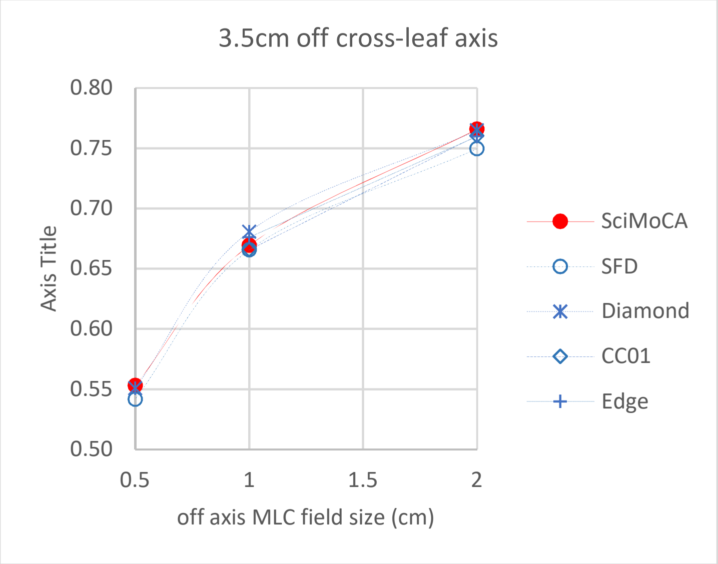
3.5cm off cross-leaf axis
Axis Title
0.80
0.75
0.70
0.65
0.60
0.55
0.50
0.5
1
1.5
2
off axis MLC field size (cm)
SciMoCA
SFD
Diamond
CC01
Edge

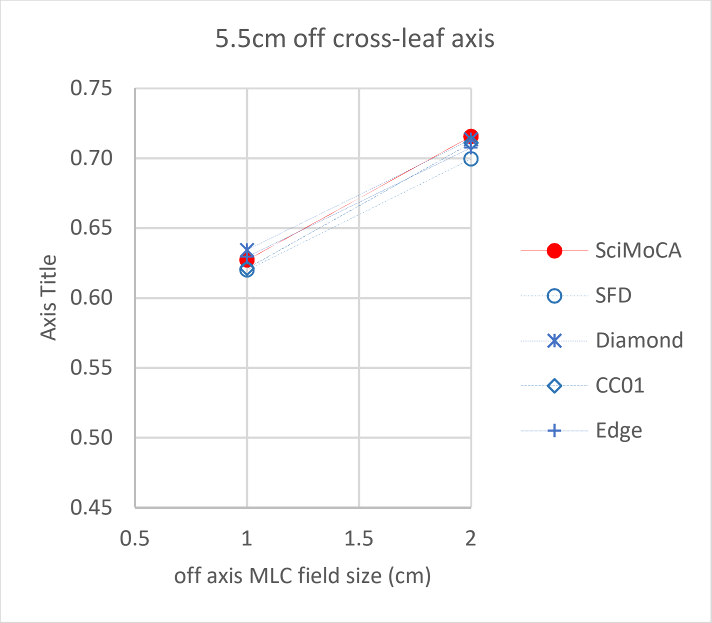
5.5cm off cross-leaf axis
Axis Title
0.75
0.70
0.65
0.60
0.55
0.50
0.45
0.5
1
1.5
2
off axis MLC field size (cm)
SciMoCA
SFD
Diamond
CC01
Edge

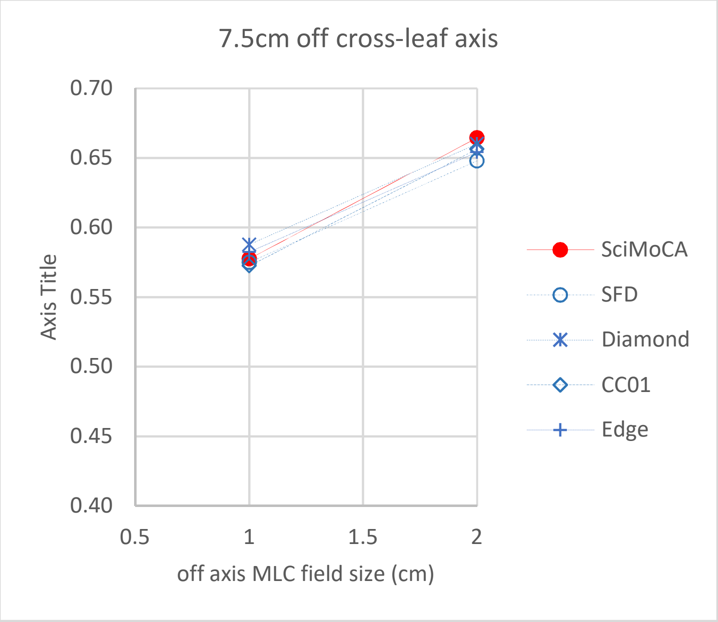
7.5cm off cross-leaf axis
Axis Title
0.70
0.65
0.60
0.55
0.50
0.45
0.40
0.5
1
1.5
2
off axis MLC field size (cm)
SciMoCA
SFD
Diamond
CC01
Edge

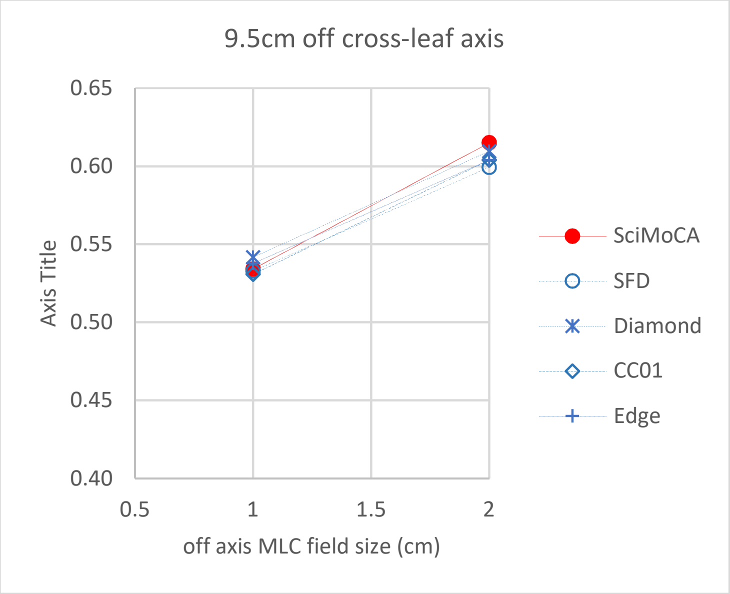
9.5cm off cross-leaf axis
Axis Title
0.65
0.60
0.55
0.50
0.45
0.40
0.5
1
1.5
2
off axis MLC field size (cm)
SciMoCA
SFD
Diamond
CC01
Edge

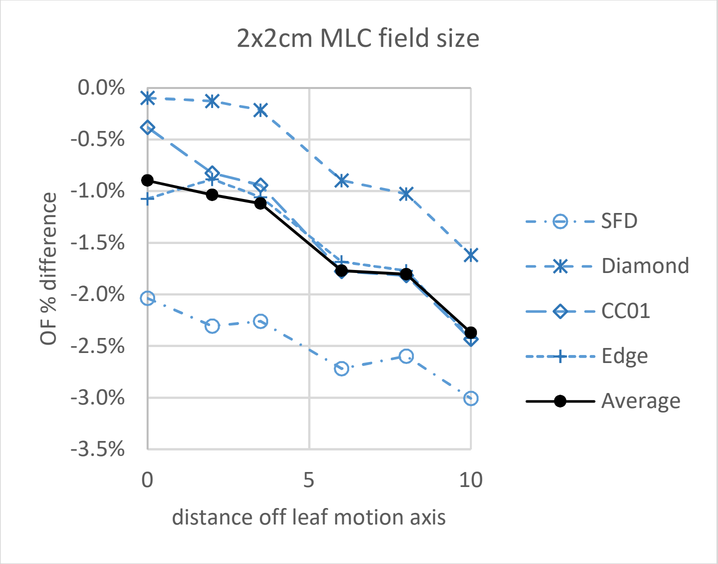
2x2cm MLC field size
OF % difference
0.0%
-0.5%
-1.0%
-1.5%
-2.0%
-2.5%
-3.0%
-3.5%
0
5
10
distance off leaf motion axis
SFD
Diamond
CC01
Edge
Average

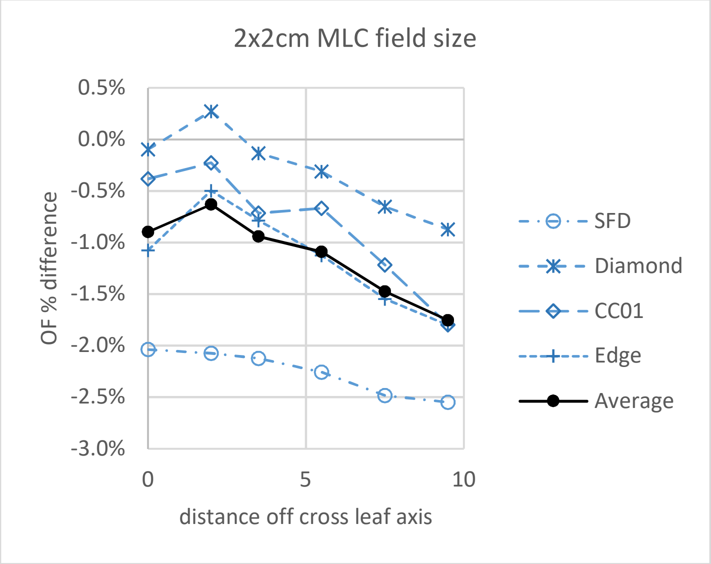
2x2cm MLC field size
OF % difference
0.5%
0.0%
-0.5%
-1.0%
-1.5%
-2.0%
-2.5%
-3.0%
0
5
10
distance off cross leaf axis
SFD
Diamond
CC01
Edge
Average

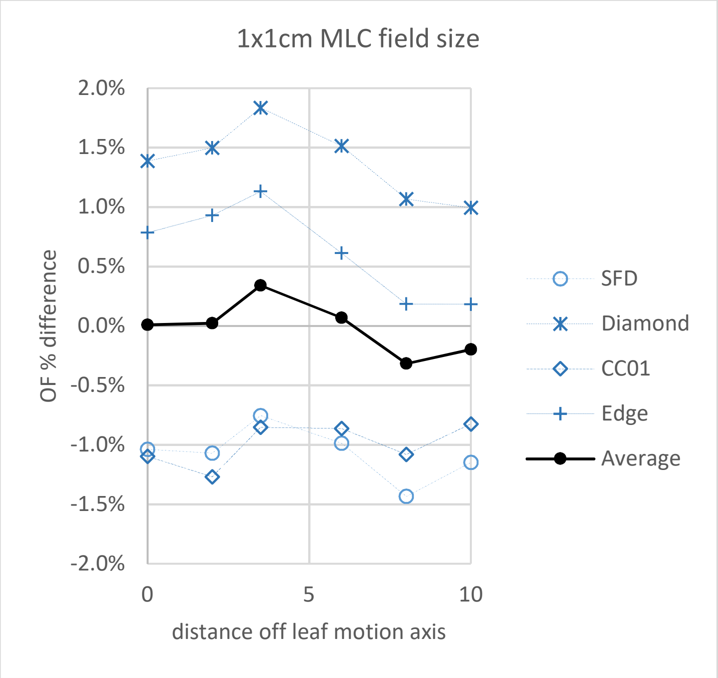
1x1cm MLC field size
OF % difference
2.0%
1.5%
1.0%
0.5%
0.0%
-0.5%
-1.0%
-1.5%
-2.0%
0
5
10
distance off leaf motion axis
SFD
Diamond
CC01
Edge
Average

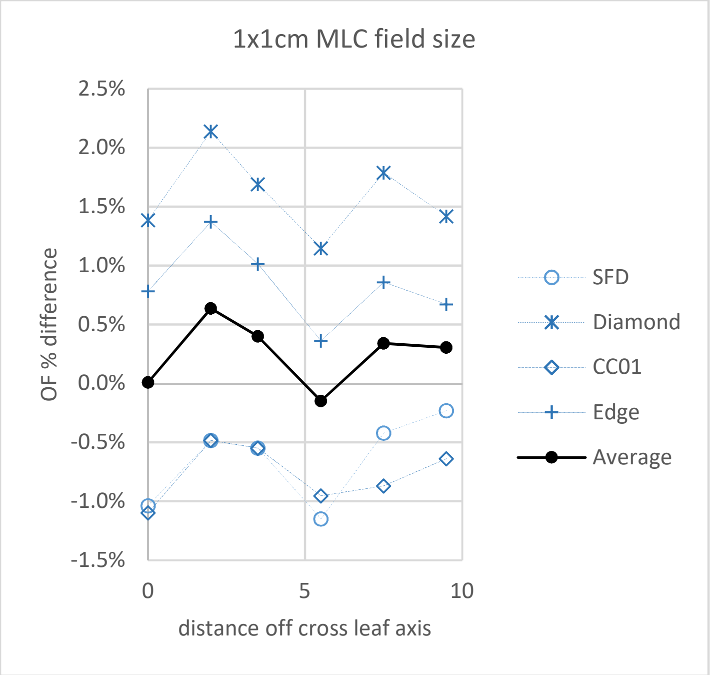
1x1cm MLC field size
OF % difference
2.5%
2.0%
1.5%
1.0%
0.5%
0.0%
-0.5%
-1.0%
-1.5%
0
5
10
distance off cross leaf axis
SFD
Diamond
CC01
Edge
Average

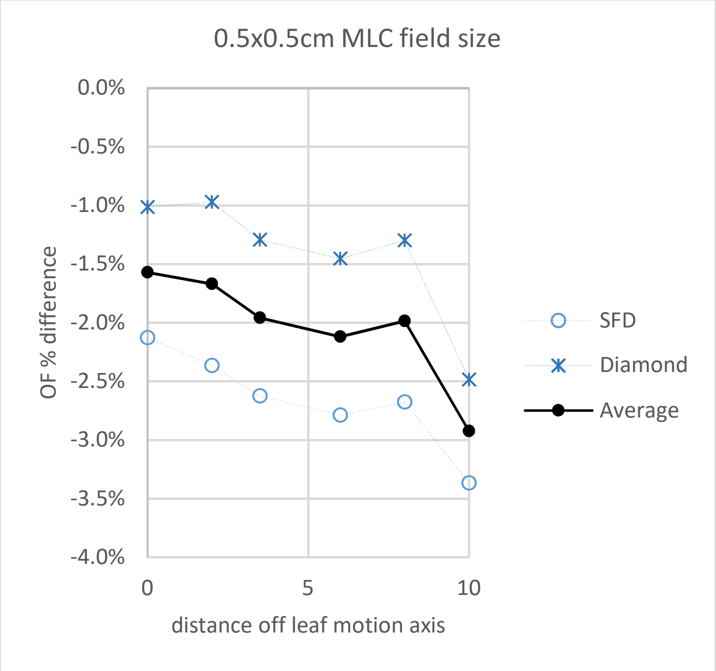

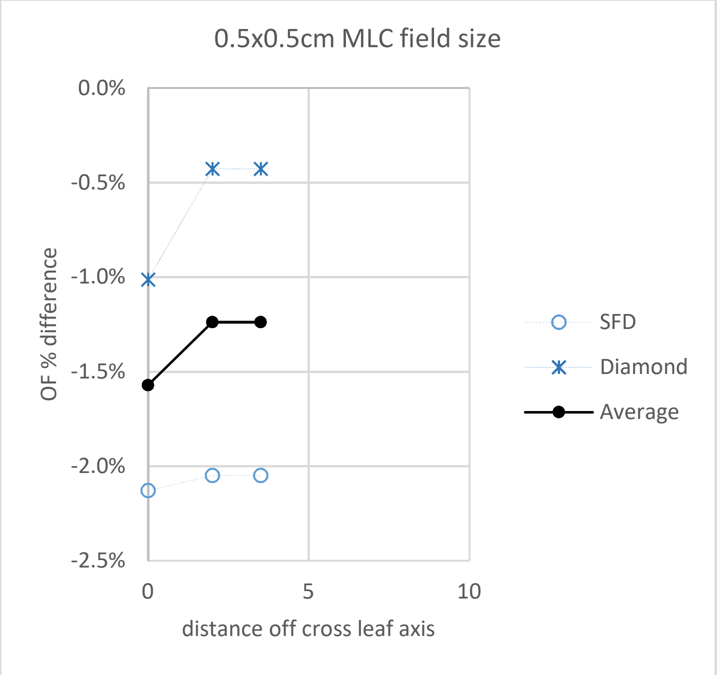

% difference between SciMoCa MC and measurement

SFD	leaf motion distance off axis (cm)						cross leaf distance off axis (cm)					
MLC	0	2	3.5	6	8	10	0	2	3.5	5.5	7.5	9.5
2x2	-2.0%	-2.3%	-2.3%	-2.7%	-2.6%	-3.0%	-2.0%	-2.1%	-2.1%	-2.3%	-2.5%	-2.5%
1x1	-1.0%	-1.1%	-0.8%	-1.0%	-1.4%	-1.1%	-1.0%	-0.5%	-0.5%	-1.1%	-0.4%	-0.2%
0.5x0.5	-2.1%	-2.4%	-2.6%	-2.8%	-2.7%	-3.4%	-2.1%	-2.0%	-2.0%			

Diamond	leaf motion distance off axis (cm)						cross leaf distance off axis (cm)					
MLC	0	2	3.5	6	8	10	0	2	3.5	5.5	7.5	9.5
2x2	-0.1%	-0.1%	-0.2%	-0.9%	-1.0%	-1.6%	-0.1%	0.3%	-0.1%	-0.3%	-0.7%	-0.9%
1x1	1.4%	1.5%	1.8%	1.5%	1.1%	1.0%	1.4%	2.1%	1.7%	1.1%	1.8%	1.4%
0.5x0.5	-1.0%	-1.0%	-1.3%	-1.5%	-1.3%	-2.5%	-1.0%	-0.4%	-0.4%			

CC01	leaf motion distance off axis (cm)						cross leaf distance off axis (cm)					
MLC	0	2	3.5	6	8	10	0	2	3.5	5.5	7.5	9.5
2x2	-0.4%	-0.8%	-0.9%	-1.8%	-1.8%	-2.4%	-0.4%	-0.2%	-0.7%	-0.7%	-1.2%	-1.8%
1x1	-1.1%	-1.3%	-0.9%	-0.9%	-1.1%	-0.8%	-1.1%	-0.5%	-0.5%	-1.0%	-0.9%	-0.6%
0.5x0.5												

Edge	leaf motion distance off axis (cm)						cross leaf distance off axis (cm)					
MLC	0	2	3.5	6	8	10	0	2	3.5	5.5	7.5	9.5
2x2	-1.07%	-0.9%	-1.1%	-1.7%	-1.8%	-2.4%	-1.1%	-0.5%	-0.8%	-1.1%	-1.5%	-1.8%
1x1	0.78%	0.9%	1.1%	0.6%	0.2%	0.2%	0.8%	1.4%	1.0%	0.4%	0.9%	0.7%
0.5x0.5												

Average	leaf motion distance off axis (cm)						cross leaf distance off axis (cm)					
MLC	0	2	3.5	6	8	10	0	2	3.5	5.5	7.5	9.5
2x2	-0.9%	-1.0%	-1.1%	-1.8%	-1.8%	-2.4%	-0.9%	-0.6%	-0.9%	-1.1%	-1.5%	-1.8%
1x1	0.0%	0.0%	0.3%	0.1%	-0.3%	-0.2%	0.0%	0.6%	0.4%	-0.1%	0.3%	0.3%
0.5x0.5	-1.6%	-1.7%	-2.0%	-2.1%	-2.0%	-2.9%	-1.6%	-1.2%	-1.2%			

8. MLC Off Axis Profiles

Small field profiles at central axis were measured at off axis distances using the IBA SFD in a water tank. These measurements were compared with the profiles calculated using the SciMoCa.

- Depth = 5 cm
- SSD = 95cm
- Field size: 1x1 cm^2 with MLCs
- Off axis distance from isocenter: 5 cm & 9 cm along leaf motion axis, 5 cm & 9 cm along cross-leaf axis, 5 cm & 9 cm diagonally (5 cm or 9 cm along leaf motion axis *and* 5 cm or 9 cm along cross leaf axis).
- Detector (in water): IBA Stereotactic Field Diose (SFD)

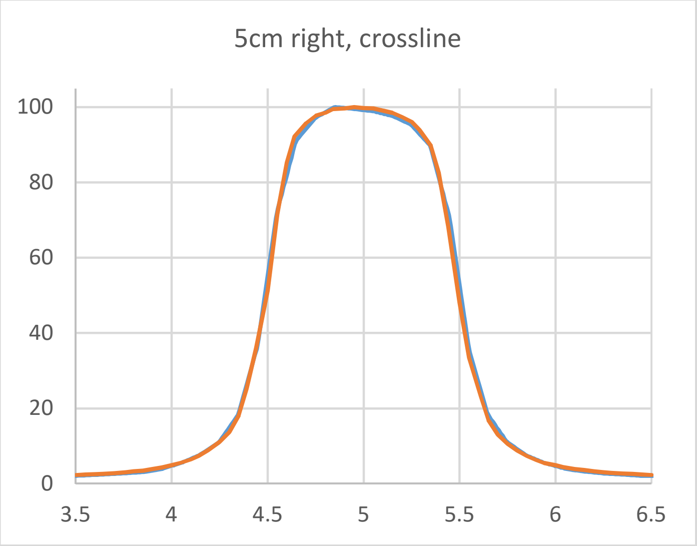

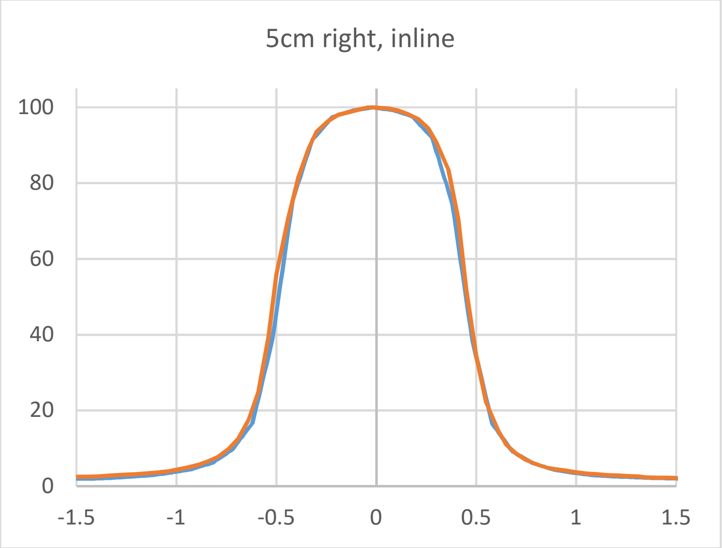

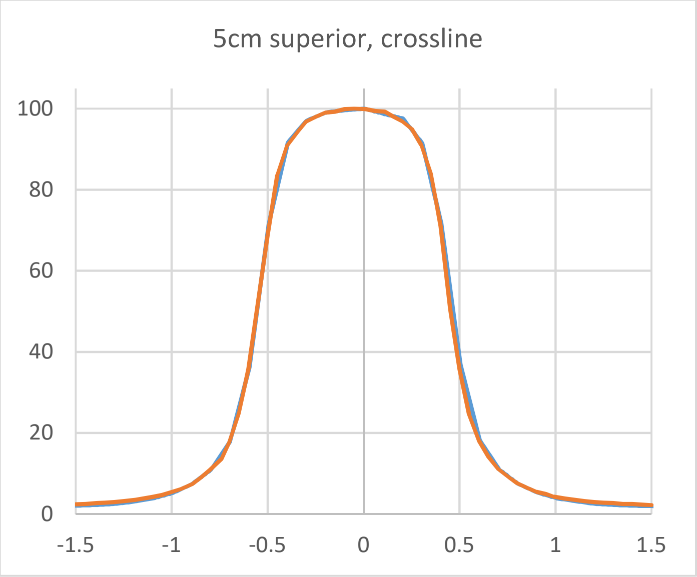

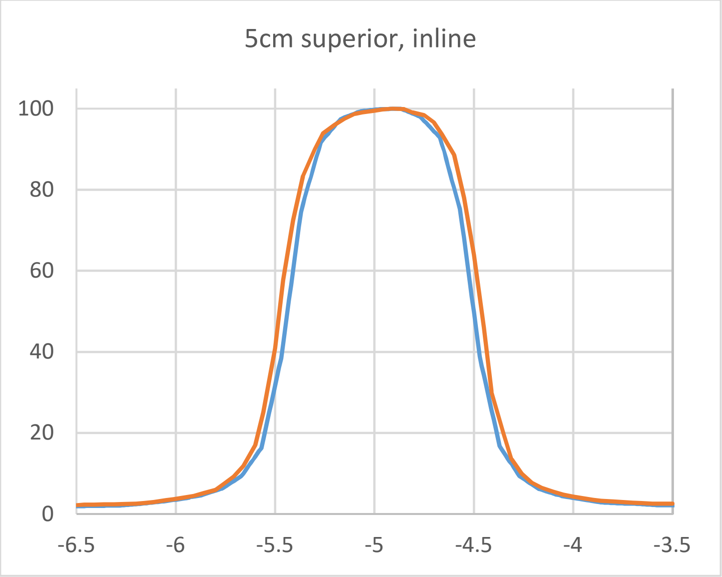

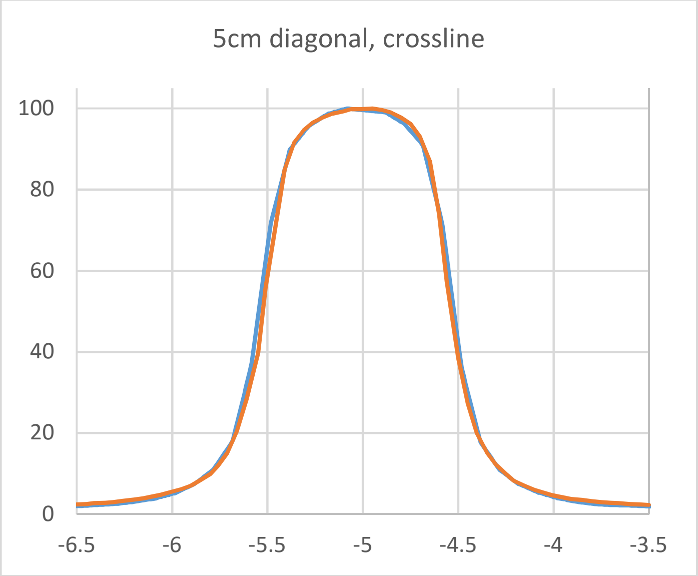
5cm diagonal, crossline
100
80
60
40
20
0
-6.5
-6
-5.5
-5
-4.5
-4
-3.5

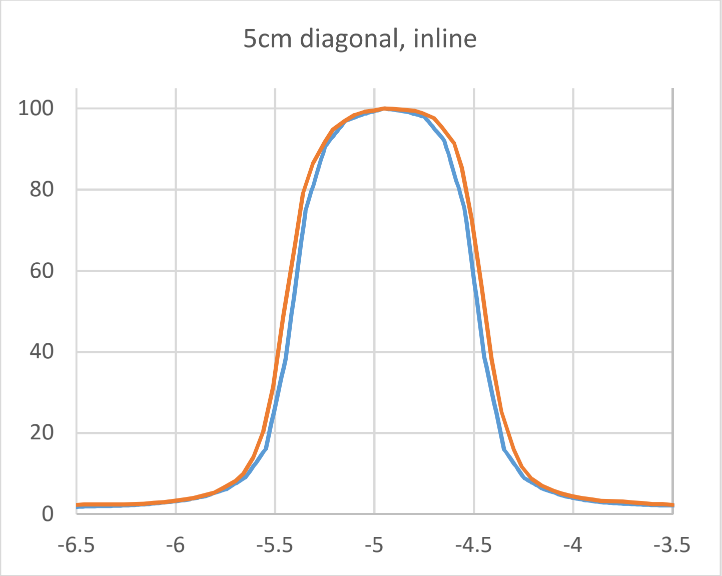
5cm diagonal, inline
100
80
60
40
20
0
-6.5
-6
-5.5
-5
-4.5
-4
-3.5

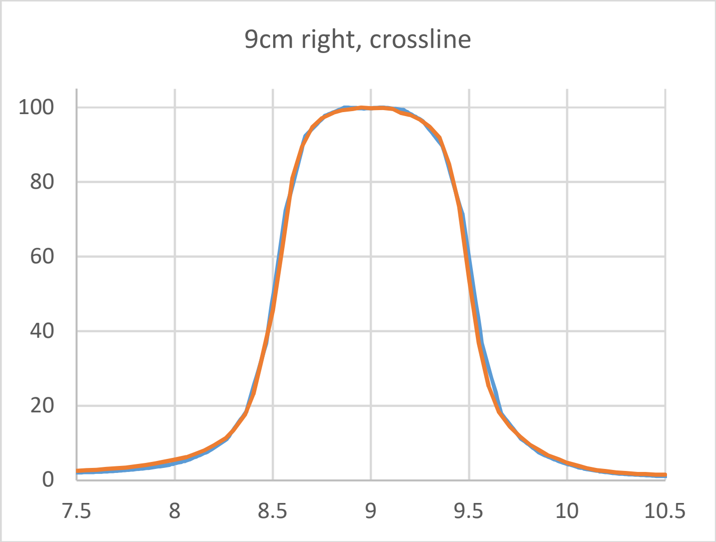
9cm right, crossline
100
80
60
40
20
0
7.5
8
8.5
9
9.5
10
10.5

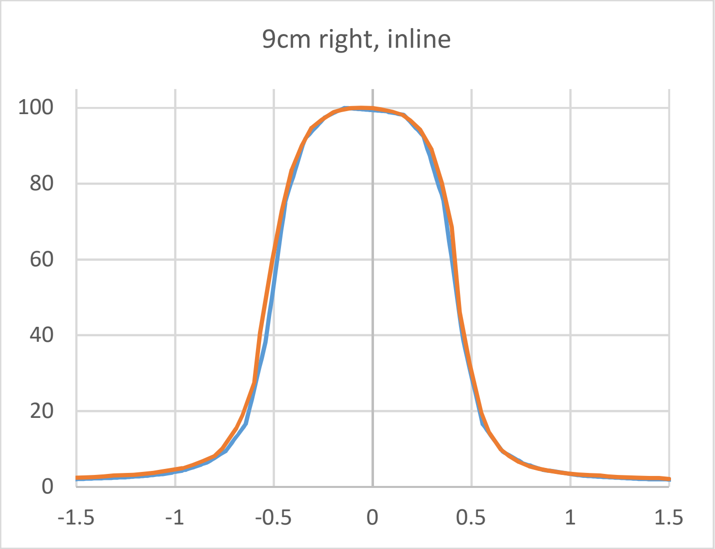
9cm right, inline
100
80
60
40
20
0
-1.5
-1
-0.5
0
0.5
1
1.5

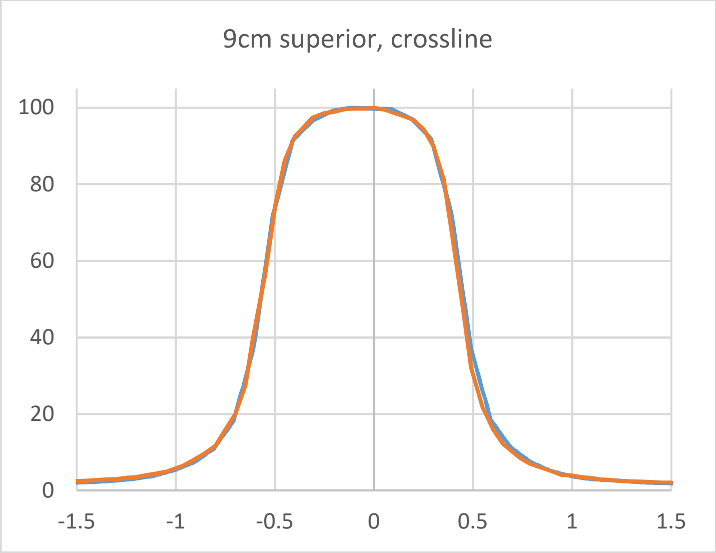
9cm superior, crossline
100
80
60
40
20
0
-1.5
-1
-0.5
0
0.5
1
1.5

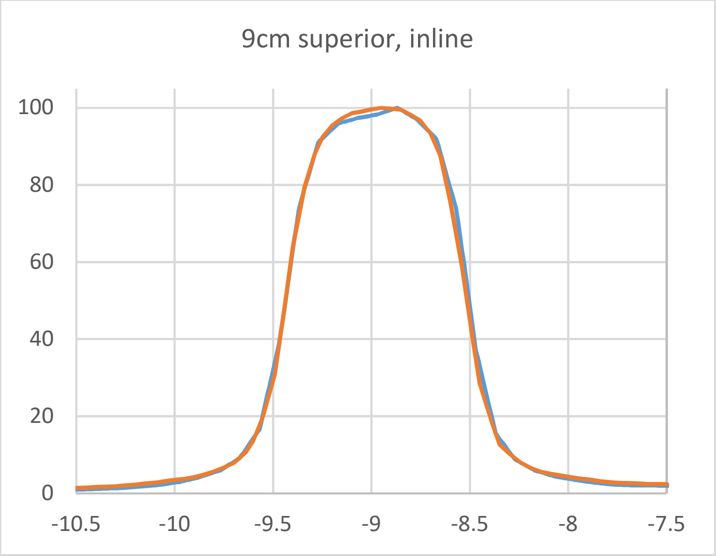
9cm superior, inline
100
80
60
40
20
0
-10.5
-10
-9.5
-9
-8.5
-8
-7.5

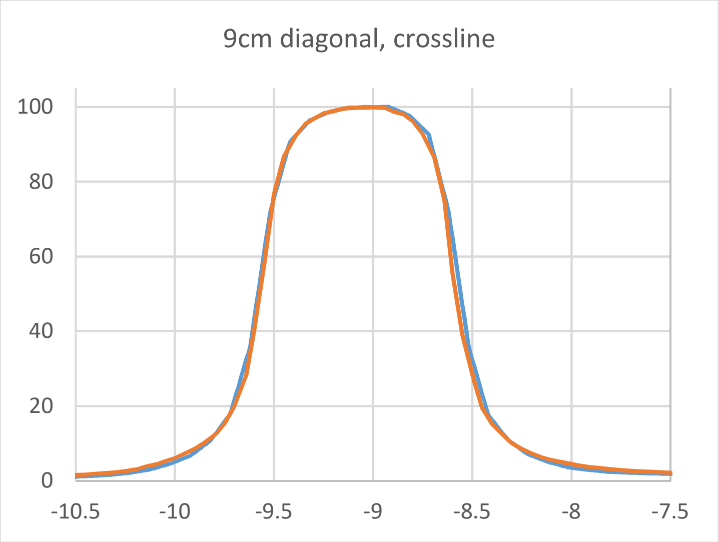

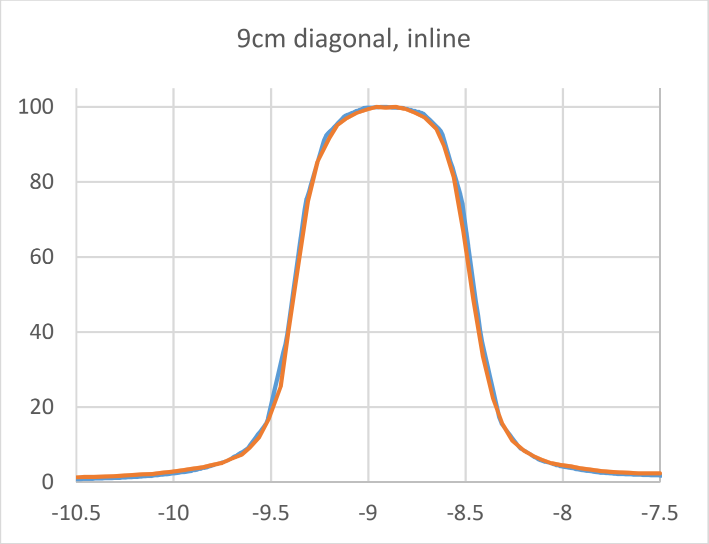

			FWHM (cm)		difference	
off axis (cm)	offset direction	scan direction	measured	SciMoCa	Cm	%
0	n/a	crossline	1.047	1.023	0.02	2.3%
0	n/a	inline	0.964	0.937	0.03	2.7%
5	right	crossline	1.000	1.021	-0.02	-2.2%
5	right	inline	0.970	0.937	0.03	3.3%
5	superior	crossline	1.009	1.024	-0.01	-1.4%
5	superior	inline	1.017	0.937	0.08	7.9%
5	diagonal	crossline	0.988	1.023	-0.04	-3.6%
5	diagonal	inline	1.012	0.938	0.07	7.3%
9	right	crossline	0.999	1.025	-0.03	-2.6%
9	right	inline	0.973	0.938	0.04	3.6%
9	superior	crossline	1.016	1.025	-0.01	-0.9%
9	superior	inline	0.923	0.933	-0.01	-1.1%
9	diagonal	crossline	0.989	1.023	-0.03	-3.4%
9	diagonal	inline	0.919	0.937	-0.02	-1.9%

Summary:

The difference in Full Width at Half Maximum (FWHM) between calculation and measurement was:

- For all profiles: 0.01cm (0.7%) ± 0.04cm (3.8%), RMS = 0.04cm (3.7%)
- For profiles at 5cm off axis: 0.02cm (1.9%) ± 0.05cm (5.0%), RMS = 0.05cm (4.9%)
- For profiles at 9cm off axis: -0.01cm (-1.0%) ± 0.02cm (2.5%), RMS = 0.02cm (2.5%)
